# Advancing precision medicines for ocular disorders: Diagnostic genomics to tailored therapies

**DOI:** 10.3389/fmed.2022.906482

**Published:** 2022-07-15

**Authors:** Priyalakshmi Panikker, Shomereeta Roy, Anuprita Ghosh, B. Poornachandra, Arkasubhra Ghosh

**Affiliations:** ^1^Grow Research Laboratory, Narayana Nethralaya Foundation, Bengaluru, India; ^2^Vitreo-Retina Services, Narayana Nethralaya, Bengaluru, India

**Keywords:** ocular dystrophies, genetic testing, gene therapy, ophthalmology, viral vectors, non-viral vectors, adeno-associated virus

## Abstract

Successful sequencing of the human genome and evolving functional knowledge of gene products has taken genomic medicine to the forefront, soon combining broadly with traditional diagnostics, therapeutics, and prognostics in patients. Recent years have witnessed an extraordinary leap in our understanding of ocular diseases and their respective genetic underpinnings. As we are entering the age of genomic medicine, rapid advances in genome sequencing, gene delivery, genome surgery, and computational genomics enable an ever-increasing capacity to provide a precise and robust diagnosis of diseases and the development of targeted treatment strategies. Inherited retinal diseases are a major source of blindness around the world where a large number of causative genes have been identified, paving the way for personalized diagnostics in the clinic. Developments in functional genetics and gene transfer techniques has also led to the first FDA approval of gene therapy for LCA, a childhood blindness. Many such retinal diseases are the focus of various clinical trials, making clinical diagnoses of retinal diseases, their underlying genetics and the studies of natural history important. Here, we review methodologies for identifying new genes and variants associated with various ocular disorders and the complexities associated with them. Thereafter we discuss briefly, various retinal diseases and the application of genomic technologies in their diagnosis. We also discuss the strategies, challenges, and potential of gene therapy for the treatment of inherited and acquired retinal diseases. Additionally, we discuss the translational aspects of gene therapy, the important vector types and considerations for human trials that may help advance personalized therapeutics in ophthalmology. Retinal disease research has led the application of precision diagnostics and precision therapies; therefore, this review provides a general understanding of the current status of precision medicine in ophthalmology.

## Introduction

Ocular dystrophies or inherited retinal diseases (IRDs) are a heterogeneous group of rare ocular diseases commonly caused by gene mutations which subsequently result in degeneration of retinal photoreceptors leading to progressive visual damage (1, 2). Around 300 genes have been recognized in which mutations can give rise to one or more of the clinical subtypes of ocular diseases (3). It is estimated that 1 in 2,000 people worldwide is affected by IRDs (4). IRDs can be familial or sporadic, syndromic or isolated, and stationary or progressive. With respect to geographic distribution, IRDs could be diffused or localized. Ocular dystrophies are inherited through all modes of inheritance i.e., autosomal dominant, autosomal recessive, X-linked, and mitochondrial (5).

Several factors have contributed to making the ocular compartment an ideal model for molecular therapies (6, 7). The tight junctions of the blood-brain barrier make the retina a fairly immune-advantaged tissue. Therefore, the normal inflammatory immune response is limited within the ocular chamber (8) as well as on the ocular surface (9, 10) due to the presence of specific molecular factors in the ocular fluids and expression of immune dampening signals on tissue surfaces that dampen the immune responses locally. This feature of the eye makes the retina relatively tolerant to the introduction of viral vectors without eliciting severe inflammatory responses (5, 11). The most advantageous feature of the eye is the low amount of vector required to obtain a therapeutic response. The possibility of extensive systemic distribution of the locally administered vector is low (12), which further inhibits undesired effects. Vector mediated gene therapy has been shown to decrease photoreceptor loss in rodent models of primary photoreceptor diseases and in dogs with a naturally occurring disease similar to human Leber’s congenital amaurosis (LCA) (13). Another beneficial feature is the ease of accessibility through intravitreal and subretinal delivery of vectors to the affected tissue of the eye (14). The differentiated and non-dividing characteristics of the retinal cells work in favor of retaining vectors with minimal loss.

Currently, there are several overlapping methods to treat ocular dystrophies under development, that not only include molecular therapies but also stem cell-based therapies and retinal prostheses (15). Novel approaches for the treatment of many eye diseases are possible today due to the successful delivery of foreign genes to ocular tissues to modify the genotype and phenotype of the cells (16). Gene therapy is one such approach that has shown success in recent years, especially in the field of ocular disease. There are over 70 clinical trials registered on clinicaltrials.gov using gene therapy for the treatment of several ocular disorders including Leber Congenital Amaurosis 2 (LCA2), Retinitis Pigmentosa (RP), Choroideremia, Leber’s Hereditary Optic Neuropathy (LHON), and Achromatopsia (Table 1), to more complex “acquired” disorders like Age-related Macular Degeneration (AMD). Improvements in viral vectors, as well as benefits of the ocular environment for gene therapy, have made this treatment modality safer and more specific, driving its acceptance in the clinic.

**TABLE 1:** Clinical trials of gene therapy using viral vectors for IRDs (ClinicalTrials.gov).

Phase	Conditions	Target gene	Interventions	Result	Sponsors and collaborators	NCT number
1/2	Achromatopsia	*CNGB3*	Subretinal rAAV2tYF-PR1.7-hCNGB3	NA	Applied Genetic Technologies Corp	NCT02599922
1/2	Achromatopsia	*CNGA3*	Subretinal rAAV.hCNGA3	NA	STZ eyetrial	NCT02610582
1/2	Achromatopsia	*CNGB3*	Subretinal AAV2/8 viral vector	NA	MeiraGTx UK II Ltd	NCT03001310
2	Choroideremia	*REP1*	Subretinal AAV-REP1	NA	Targeted Genetics Corporation	NCT02407678
2	Choroideremia	*REP1*	Subretinal AAV2-REP1	NA	Byron Lam	NCT02553135
2	Choroideremia	*REP1*	Subretinal rAAV2.REP1	NA	STZ eyetrial	NCT02671539
1/2	Choroideremia	*REP1*	Subretinal rAAV2.REP1	Improved visual acuity at 6 months	UK Department of Health and Wellcome Trust	NCT01461213
1/2	Choroideremia	*REP1*	Subretinal rAAV2.REP1	NA	Ian M. MacDonald, Alberta Innovates Health Solutions	NCT02077361
1/2	Choroideremia	*CHM*	Subretinal AAV2-hCHM	NA	Spark Therapeutics	NCT02341807
1/2	LCA	*RPE65*	Subretinal tgAAG76 (rAAV 2/2.hRPE65p.hRPE65)	Moderate and temporary improvement of retinal sensitivity	National Institute for Health Research and others; Targeted Genetics Corporation	NCT00643747
1	LCA	*RPE65*	Subretinal rAAV2-CBSB-hRPE65	Improved visual sensitivity at 3 and 12 months followed by gradual diminution over 6 years, no serious adverse event	University of Pennsylvania, National Eye Institute (NEI)	NCT00481546
1	LCA	*RPE65*	Subretinal AAV2-hRPE65v2	Modest improvement in subjective vision up to 2 years. The greatest improvement was noted in children. 1 of 12 subjects had temporary macular hole, no other adverse events. Greatest improvement in young patients.	Spark Therapeutics, The Children’s Hospital of Philadelphia	NCT00516477
1	LCA	*RPE65*	Subretinal rAAV2-hRPE65	NA	Hadassah Medical Organization	NCT00821340
1	LCA	*RPE65*	Subretinal AAV2/5 OPTIRPE65	NA	MeiraGTx UK II Ltd.	NCT02781480
1/2	LCA	*RPE65*	Subretinal AAV2/5 OPTIRPE65	NA	MeiraGTx UK II Ltd., Syne Qua Non-Limited	NCT02946879
1/2	LCA	*RPE65*	Subretinal AAV2-hRPE65v2 (contralateral eye treatment)	Contralateral eyes of 11 subjects from 12 subjects who were enrolled in the previous phase 1 trial. Improved mobility and light sensitivity from day 30 to year 3. One case of endophthalmitis	Spark Therapeutics, The Children’s Hospital of Philadelphia	NCT01208389
3	LCA, RP	*RPE65*	Subretinal AAV2-hRPE65v2	Bilateral subretinal injection. Significant improvement in functional vision as measured by the change in mobility testing between baseline and one year	Spark Therapeutics	NCT00999609
½	LCA	*RPE65*	Subretinal rAAV2-CB-hRPE65	NA	Applied Genetic Technologies Corp	NCT00749957
½	LCA	*RPE65*	Subretinal rAAV2/4.hRPE65	NA	Nantes University Hospital	NCT01496040
½	LHON	*ND4*	Intravitreal rAAV2-ND4	Improved visual acuity and enlarged visual field, No local or systemic adverse events	Bin Li	NCT01267422
3	LHON	*ND4*	Intravitreal GS010 (AAV2/2 ND4)	NA	GenSight Biologics	NCT02652767
3	LHON	*ND4*	Intravitreal GS010 (AAV2/2-ND4)	NA	GenSight Biologics	NCT02652780
½	Retinoschisis	*RS1*	Intravitreal AAV8 scRS/IRBPhRS	NA	National Eye Institute (NEI)	NCT02317887
½	Retinoschisis	*RS1*	Intravitreal rAAV2tYF-CB-hRS1	NA	Applied Genetic Technologies Corp	NCT02416622
1	RP	*MERTK*	Subretinal rAAV2-VMD2-hMERTK	NA	Fowzan Alkuraya	NCT01482195
½	RP	*Channelrhodopsin-2*	Intravitreal RST-001	NA	RetroSense Therapeutics	NCT02556736
1/2	RP, X-linked	*RPGR*	Subretinal AAV-RPGR	NA	NightstaRx Limited	NCT03116113
½	Stargardt Disease	*ABCA5*	Subretinal SAR422459, Lentiviral vector	NA	Sanofi	NCT01367444
½	Stargardt Disease	*ABCA4*	Subretinal SAR422459, Lentiviral vector	NA	Sanofi	NCT01736592

There remains a myriad of challenges that will need to be addressed in order to attain the long-term success for different treatment modalities such as tissue specific expression, long term sustenance of the therapy, limiting immune reactions, reducing prices, and improving accessibility. Currently, an approved treatment is Voretigene neparvovec-rzyl, a viral vector mediated gene therapy approved for *RPE65*-mediated IRD, which accounts for about 2% of autosomal recessive RP and 16% of LCA (17, 18). The heterogeneous nature of IRDs makes it difficult for the development of a common treatment for a wide number of patients (5). Most critically, the costs of current gene therapies are substantial, with large pharmaceutical initiatives being cautious about the field given the limited number of individuals that could significantly benefit from such tailored therapies.

In this review, we emphasize the most advanced methodologies to recognize new genes and variants associated with diverse ocular disorders and the complexities linked with them using a few examples from existing literature. We also explore the strategies, challenges, and potential of gene therapy for the treatment of IRDs. Further, we discuss the multiple aspects of gene therapy which can help to improve personalized therapeutics in ophthalmology.

## Genomic medicine for the retina: Genes associated with inherited and acquired retinal disorders

Current progress made in genomics has led to the identification of new genes and variants responsible for a host of inherited and age-related ocular disorders. The evolving molecular research studies have revealed the genetic underpinnings and the disease mechanism of such diseases. As a result, scientists have outlined many genes and their variants that can impact the vision and health of our eyes. For example, the genomic revolution revealed the genetic causes of LCA, an IRD which leads to extreme vision loss in childhood, and AMD, a common cause of blindness in the elderly (19). Presently, more than 20 LCA genes have been identified and documented (20). Nowadays, genetic testing can be performed quickly in many kinds of retinal diseases and often aids diagnosis. More importantly, gene-specific treatments have also been tested clinically. AMD is a complex disease caused by a combination of genetics and environmental factors but despite its complexity, more than 40 loci have been accounted for 15 to 65% of AMD pathology (20). Based on the genetic findings, it is evident that an early diagnosis through genetic testing can help evaluate patients’ conditions for deciding on the treatment plan(s) and follow-up care to avoid or delay irreversible vision loss (21). Today it is accepted that genetics play a significant role in the causation and progression of ocular disorders; a few of which are listed in Table 2 and briefly covered in this review.

**TABLE 2:** Genes linked to human eye disorders.

Sl. no	Disease	Gene/Variant
1	Retinitis pigmentosa	*MERTK, RPGR, PDE6B, PRPF3, HK1, RHO, AGBL5*, etc.
2	Stargardt’s disease	*ABCA4*, etc.
3	Inherited optic neuropathies	*OPA1, RPE65, Complex I, ND1, ND4* or *ND6* genes, etc.
4	Achromatopsia	*CNGB3, CNGA3*, *GNAT2, PDE6C, PDE6H*
5	AMD	*ABCR/ABCA4, CFH†, CST3, ERCC6, FBLN5, NOS2A, CFH, CF, C2, C3, CFB, HTRA1/LOC, MMP-9, TIMP-3, SLC16A8, FBLN6 (HMCN1), HTRA1†, LOC387715/ARMS2, RAXL1, TLR4, ERCC6, FBLN5, HMCN1, HTRA1, RAX2*, etc.
6	Leber congenital amaurosis type 10 (LCA10)	*CEP290*
7	Leber congenital amaurosis type 2 (LCA2)	*RPE65*
8	X-linked retinoschisis	*RS1*
9	Glaucoma	*CALM2, MPP-7, Optineurin, LOX1, CYP1B1, CAV1/2, MYOC, PITX2, FOXC1, PAX6, CYP1B1, LTBP2*, etc.
10	Cataract	*GEMIN4, CYP51A1, RIC1, TAPT1, TAF1A, WDR87, APE1, MIP, Cx50/GJA3 & 8, CRYAA, CRYBB2, PRX, POLR3B, XRCC1, ZNF350, EPHA2*, etc.
11	Marfan syndrome	*FBN1, TGFBR2, MTHFR, MTR, MTRR*, etc.
12	Myopia	*HGF, C-MET, UMODL1, MMP-1/2, PAX6, CBS, MTHFR, IGF-1, UHRF1BP1L, PTPRR, PPFIA2, P4HA2*, etc.
13	Diabetic retinopathy	*AKR1B1 (ALR2)*, a*2*b*1 integrin gene, bFGF, EPO, HFE, Mn-SOD, IGF-I, ICAM-1, PON1, PPARGC1, UCP2*, etc.
14	Usher syndrome	*MYO7A*
15	Uveal melanoma	*PTEN, BAP1, GNAQ, GNA11, DDEF1, SF3B1, EIF1AX, CDKN2A, p14ARF, HERC2/OCA2*, etc.
16	Choroideremia	*CHM*
17	Polypoidal choroidal vasculopathies	*C2, C3, CFH, SERPING1, PEDF, LOC387715, CETP, ARMS2-HTRA1, FGD6, ABCG1*, etc.

## Precision diagnostics: Clinical genetic testing for ocular disorders

**FIGURE 1:**
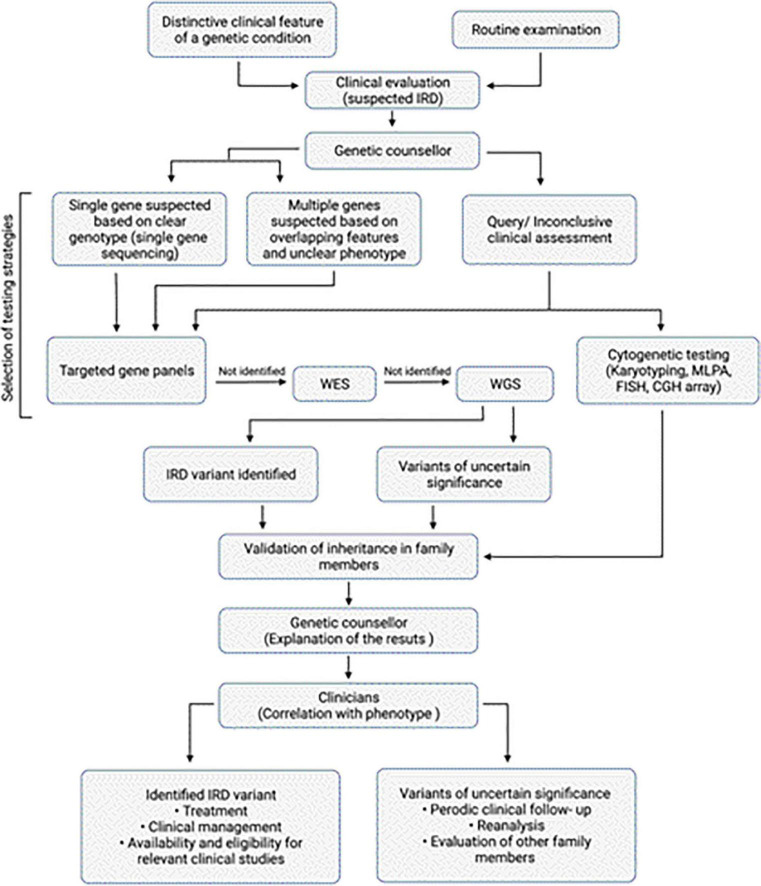
Overview of diagnosis of IRDs. Algorithm for clinical and genetic assessment and diagnosis of IRDs. The work flow also depicts the various genetic testing approaches that can be selected based on the clinical assessment.

As we are entering the age of genomic medicine, advances in genetic research can now provide precise and robust diagnoses. Genetic evidence today can provide information regarding prognosis from the evolving body of genotype-phenotype correlations and protein function, which can then help in directing precise therapeutic interventions (22). This has been further bolstered by the rapid advancement in DNA sequencing methodologies and analysis tools. In the field of IRDs in particular, the impact of such advances is evident. With mutations in more than 300 genes implicated in IRDs, along with several other modifying elements, the genetic complexities of IRDs are evident (23). Genetic screening of IRDs comes with its set of challenges particularly as a proportion of screened patients have genomic alterations which have not yet been established as causative or pathogenic in the literature (24). Identification of disease-causing gene variant(s) is extremely important to better understand the disorder and its inheritance. The importance of establishing a genetic diagnosis, so patients can get access to the latest treatments options has become evident with the approval of first gene therapy for IRDs caused by biallelic variants in the *RPE65* gene (25) as well as with the rise in many more gene-based treatments (Figure 1). Additionally, due to considerable genetic and phenotypic heterogeneity it may be difficult to attribute a particular disease-causing gene unless functional molecular pathways are confirmed. The availability of comprehensive genomic diagnostic techniques today (Table 3) has improved our access to personalized medicine and will be discussed in the following sections. Next generation sequencing (NGS) and cytogenetic testing are two main clinical genetic tests primarily considered for diagnosis of any IRD. To establish genotype–phenotype correlations and to better understand the disease, it is important to retrieve a molecular diagnosis which will help in determining a prognosis. For most genetic eye conditions, sequencing of either a single gene, such as *PAX6* for aniridia in adults, or targeted gene panels, such as for retinal dystrophies, is usually considered as the initial route of molecular analysis (26). Furthermore, alternative genetic testing methods like genome wide copy number variant (CNV) analysis by microarray may be more suitable for syndromic conditions.

**TABLE 3:** Advantages and limitations of various genetic testing approaches.

Technique	Advantages	Limitations
**NGS**		
Target gene panels	Identifies variants in specific regions (exons and flanking introns) Rapid analysis Customizable Low cost	Variants limited to selected/known genes Cannot identify structural variants Needs frequent updates- time consuming and costly
WES	Identifies variants in all protein-coding regions Lower cost compared to WGS	Cannot detect structural variants or deep intronic variants Requires exome capture or enrichment methods during library preparation
WGS	Identifies variants in the entire genome Detects rearrangements and structural variants, deep intronic variants, CNV	Requires long and complex analysis Expensive Very large volume of data generated
**Cytogenetic tests**		
MLPA	Detects small rearrangements, upto 40 targets High throughput and low cost	Problems with mosaicism Cannot detect heterozygosity
FISH	Detects balanced rearrangement and mosaicism Can quantify multiple copies	Cannot detect small rearrangements Limited number of targets Cannot detect heterozygosity
CGH array	Detects very small rearrangements (100 kb–5 mb) Entire genome can be probed	Cannot detect heterozygosity Low throughput Costly equipment and reagents
Quantitative/Sq- PCR	Detects small rearrangements and point mutations Can quantify multiple copies Low cost	Test optimization and efficiency a concern Limited number of targets

### Next generation sequencing

Next generation sequencing is usually the foremost approach for examining of genetically heterologous eye disorder. It allows massive parallel sequencing of multiple targets from multiple samples (27). It involves sequencing of short DNA fragments and then aligning them to a reference genome, followed by identification of the variation and annotation before the final analysis. NGS methods include targeted gene panels or clinical exome, whole exome sequencing (WES) and whole genome sequencing (WGS). Illumina, Ion Torrent, Complete Genomics Technology, Third Generation Sequencing (3GS; PacBio and Oxford Nanopore) are examples of few NGS platforms that are being used routinely. The average turnaround time for most of the gene panel-based test is between 2 and 4 weeks depending on the service provider and the size of the panel. Further, within the same sequencing platform, different devices provide different levels of throughput, e.g., Illumina sequencing machines include the MiniSeq, MiSeq, NextSeq, NovaSeq, and HiSeq models. The MiniSeq gives 7.5 Gb with 25 million reads/run at 2 × 150 bp reads; MiSeq can perform 2 × 300 bp reads, 25 million reads for an output of 15 Gb; NextSeq can provide 120 Gb with 400 million reads at 2 × 150 bp read length (28). The Ion Torrent system from Thermo-Fisher includes Ion Personal Genome Machine™ (PGM™) System, Ion Proton™ System, Ion S5 system and ION S5 XL system, each with different throughput features (29). In contrast to second generation sequencing methods (Illumina and Ion torrent), third generation sequencing methods provide longer reads for DNA (and RNA) molecules, e.g., Pacific Biosciences (PacBio) which has two sequencing systems, the RSII and Sequel. Such a sequencing process, also known as SMRT (Singe Molecule Real Time) sequencing, can sequence very long fragments, up to 10–50 kb and 350 megabases of sequence per SMRT cell. The new systems can generate ∼365,000 reads, with average reads of 10–15 kb (7.6 Gb of output) (28). The costs for sequencing include many components apart from consumables and instrument, which are the labor and the bioinformatics pipeline at the end of the process. It is therefore important to understand which technology is best suited for specific genomics applications based on the questions posed and the clinical information available for specific cases.

#### Targeted gene panels

Targeted sequencing typically captures the smallest amount of genetic information and is becoming increasingly valuable since they are customizable. Such approaches utilize DNA capture and enrichment method that specifically focus on a “virtual gene panel” targeting the analysis on certain select subset of genes (30–32). The selection of genes is typically based on currently existing genotype-phenotype knowledge, gene discoveries, and functional knowledge of molecular pathways involved in disease pathology. These panels can be custom designed for specific target exons as well as flanking introns of the genes that are known to be associated with certain genetic eye disease. For example, Oculome, a targeted gene panel, was designed to screen 429 known eye-related disease-causing genes (30) specifically ocular birth defects and inherited eye conditions. These have five overlapping sub-panels for several anomalies including congenital cataract and lens- associated conditions (70 genes), glaucoma (59 genes), micropthalmia-anopthalmia-colomoba (86 genes) retinal dystrophies (235 genes), and albinism (15 genes) (30). It provided a definitive diagnosis with 25% diagnostic rate in a study of 277 patients. It was developed with the goal of maximizing the chances of detecting pathogenic mutations with a single genetic test. Similarly, several genetic panels have been designed to cover known disease associated retinal genes. Another group designed a panel covering 176 retinal genes (called NGS176) and obtained a molecular diagnosis for 54.9% of the patients from a study of 488 patients. Their idea was to develop a first-tier genetic test for most IRD patients with strong diagnostic yield (33). In another panel, coverage of 214 disease-associated genes included exons, flanking introns and 5’- or 3’-untranslated regions with specific deep intronic regions. A disease-causing variant was found in 51% of cases out of 192 patients tested (32). Due to recent detection of many population-enriched pathogenic variants, the customizable element of such genetic panels is becoming highly valuable. Recently in Japan, in a RP cohort, *EYS* gene variants we found to be the causative in 51% of the cohort (34). Similarly, several other studies have associated such specific variants/mutations to certain population, like, *RAX2* in Belgium (35), *RP1* (36), and *ABCA4* (37) in Spain, and *PDE6B* in a Jewish community in Caucasia (38). Such associations direct the design of more population-specific genetic panels as well. The development of gene panels evolves from the accumulation of contemporary knowledge of the molecular functions and their link to the clinical pathologies. The use of targeted gene panels allows for maximum coverage of relevant genomic regions and genes in a cost-effective manner. However, these panels need to be updated frequently, which is one of their biggest limitations. Every time a novel gene or variant associated with a particular genetic eye disorder is identified, the panel must be redesigned which involves time and cost, leading to infrequent updates (Table 3). In such scenarios, “virtual” gene panels could be far more efficient due to quicker bioinformatic refreshing.

#### Whole exome sequencing

Whole exome sequencing involves selection, enrichment, and sequencing of exons of known protein coding genes. Although the exome is only 1.5% of the genome, most of the disease-causing gene have been found to be within the protein coding sequence (39, 40). Since all exons are covered by WES, it enables the variants to be detected even if they are not fully elucidated (41–43). Additionally, such information also allows for future investigation of such data when new genes are discovered. However, WES has its own limitation including the inability of detecting deep intronic sequences, or inversions, translocations, and non-coding rearrangements. As the amount of data generated by WES is large, it has the potential to provide information for the future resolution on unsolved diagnosis.

#### Whole genome sequencing

Whole genome sequencing far exceeds the coverage offered by gene panels and WES. It enables coverage of PCR intractable genomic regions including GC rich regions (44–48). WGS allows for identification of variants such as CNV, deep intronic variants, structural variations by allowing coverage of the entire genome (49–51). However, due to this the cost associated with WGS as well as the analysis and interpretation, it is far more expensive than the other methods. WGS mainly becomes valuable for cases unsolved by targeted screening.

### Cytogenetic testing

Apart from the tests mentioned above, cytogenetic testing is also widely used to detect chromosomal abnormalities, CNVs as well as to verify NGS findings (52). Various techniques are a part of cytogenetic testing including fluorescent *in situ* hybridization (FISH), karyotyping, qualitative fluorescent polymerase chain reaction (QF-PCR) and microarray-based comparative genomic hybridization (array-CGH) and multiplex ligation-dependent probe amplification (MLPA) assay (Table 3).

Fluorescent *in situ* hybridization is usually used to detect the presence or absence of a specific DNA sequence on a chromosome using sequence specific fluorescent probes, especially for disorders like ocular lymphoma and melanoma (53, 54). On the other hand, one of the most conventional ways of testing chromosomal abnormalities is karyotyping, which detects large chromosomal anomalies (5–10 kb) including deletions, duplications, and inversions (55, 56). Many ocular conditions such as presenile cataract, glaucoma, corneal ectasias, nystagmus, strabismus, and retinovascular anomalies are commonly found with Down’s syndrome which can easily be detected by karyotyping (57–59). Contrary to this, array-CGH is a more detailed and sensitive technique which helps determine abnormalities ranging from 100 kb–5 mb, by analysing CNVs (52, 60). It has been shown that this method is a preferred initial genetic test for patients with syndrome-related ocular diseases due to its high detection rates for such cases. To rule out Wilms tumour, aniridia, genitourinary anomalies and intellectual disability (WAGR) syndrome, array-CGH is commonly used to detect mutation involving the *WT1* and *PAX6* genes in children with aniridia (26, 61). Apart from this, QF-PCR is another technique used to quantify and confirm copy number in a specific region by amplifying specific regions of DNA. It can also detect common aneuploidies (62). In general, CNVs are better detected by array-CGH when compared to FISH and QF-PCR. However, the results provided by this method usually needs further validation by other quantitative PCR methods (63). One of the methods being used to validate array-CGH is MLPA assay, which can detect CNVs of specific genes including small intragenic rearrangements (64). A study analysing mutations in *PAX6* gene showed that MLPA enhanced the molecular diagnosis of aniridia (65). Out of 70 individuals affected with aniridia, 24 different point mutations in the *PAX6* gene were identified in 34 patients after sequencing. In additional eight patients, MLPA identified deletion of one or more exons of *PAX6* gene, demonstrating the necessity to screen for larger deletions in the gene in addition to sequencing of exons. Combinations of sequencing and MLPA are used routinely for the ocular pediatric tumor, Retinoblastoma (66).

### Access, availability and challenges of genetic testing for personalized therapeutics

Sequencing technology has evolved dramatically that has helped reduce the time and cost of sequencing entire genomes or sections of genomes in a targeted fashion. This is due to epochal developments of instruments and sequencing chemistries that has improved time, efficiency and accuracy. While the process of sequencing today takes a few hours, the initial efforts for sequencing had taken years. Previously, genetic testing has been recommended by IRD specialists and ocular genetic counselors at large academic research centers (67–69). However, this approach could not meet patient demand for three important reasons. Firstly, due to the scarcity of IRD specialists and ocular genetic counselors at academic medical centers to meet patient demand for genetic testing (70). Even though there are approximately 5,000 certified genetic counselors in the United States, less than 1% of them are proficient in ophthalmology (71). For a provisional clinical diagnosis, it is important for the affected individual to be screened by a retina specialist who has expertise in IRDs. Secondly, many individuals suffering from IRDs are geographically secluded from those centers and are either unwilling or unable to travel. Presently, community-based retina specialists in partnership with teleconference-based genetic counselors are helping to disclose results to individuals and are managing conversations regarding complex results and risks to family members (72). These telemedicine-based genetic counseling services are becoming more extensively available to assist geographically or economically disadvantaged individuals in accessing specialists in ocular genetics. Lastly, the use of genetic testing worldwide is still restricted due to budget constraints and the facility of regional healthcare systems to cover the cost of genetic testing, particularly in developing and underdeveloped countries with poor resources (73).

Genetic testing helps in molecular diagnosis that makes individuals access to the latest treatment options (Figure 1). In many IRDs, the vast degree of variability and reduced penetrance complicate the result interpretation for subjects in early stages of disease (74, 75). In such cases, carrier testing may be helpful. Carrier testing is performed on individuals who are asymptomatic but may have a mutated allele for a genetic disease that can be passed on to the next generation. This test identifies individuals carrying a single pathogenic variant in a recessive or X-linked disease gene which when present with another pathogenic variant can cause genetic disease (74). In the past, genetic testing was performed on a single gene basis where a small number of genes, closely associated with the disease on the basis of clinical evaluation were tested (67). With the introduction of NGS, testing multiple genes in a single assay has become possible (76). Before performing genetic testing, an IRD specialist or an ocular genetic counselor familiar with the genetics of the various retinal diseases should educate the affected individuals and their caregivers regarding the benefits, limitations, and potential implications of genetic testing (77, 78). For example, due to phenotypic overlap among various IRDs, targeted genetic testing may miss some differential diagnoses (76). Contrarily, broader testing strategies increase the possibility of unexpected or unclear results and the conditions that seem isolated may actually be syndromic. It is crucial for individuals to understand that genetic testing doesn’t guarantee a molecular diagnosis for their IRD since not all the variants and genes linked to IRDs have been identified (77). Therefore, testing may not identify the disease-causing variant for all individuals and the efficiency of similar testing strategies across different ethnicities may yield varying efficiencies (79). Therefore, efforts are now underway to sequence genomes across various ethnicities and enhance the reference genome sequences for under-represented groups (80). Usually, if a variant does not fulfill the pathogenicity criteria, or the function of the variant has not been experimentally determined, then it is classified as a variant of uncertain significance (VUS) (81). Sometimes, variant interpretation can be challenging even in the presence of a clear phenotype since in IRDs, mutations in different genes may yield the similar retinal pathologies. It is important to be cautious when reporting the VUS and often additional testing or reanalysis of the data in light of emerging new functional information is recommended to assess the effects of these variants. Moreover, there is a possibility that with advances in knowledge and relationship between variants and disease pathology, the VUS could later be identified as pathogenic. Early and precise diagnosis is important for individuals with IRDs to facilitate patient decision-making, recognize suitable clinical studies or treatment opportunities, and improve patient outcomes (Figure 1).

## Tailored therapeutics: Inherited retinal diseases, ideal targets for gene therapy

Over the last decade, extensive research has been done on gene transfer techniques for treating ocular diseases through gene therapy. The eye is easily accessible for topical or localized drug delivery including direct injection of gene therapy vectors. A variety of such vectors have now been tested for retinal applications that can be engineered for the delivery of therapeutic genes to ocular tissues for the treatment of many eye diseases (82). In the majority of IRDs the defective genes have an effect on the retinal pigment epithelium (RPE), the photoreceptor layers and underlying choriocapillaris (83). The availability of animal models that are genetically well-defined and the ease of gene delivery to the retina and vitreous has helped rapidly advance research on ocular gene therapy (84). The direct ocular delivery can limit immune responses toward the vector and transgene, with the blood-retinal barrier helping to restrain the systemic spread (5). The non-dividing stable cells of the retina are conducive to the use of a variety of vector types to produce sustained transgene expression with the goal of vision recovery. However, the differential progression rates of different IRDs provides challenges in identification of therapeutic windows during which rectification of the gene defect may prevent further damage.

The most common IRDs are RP, LCA, choroideremia, LHON, Achromatopsia, Stargardt disease, and X- linked retinoschisis (XLRS) (83). Thus, gene therapies in development for ocular diseases focus on these diseases alongside more common retinal vascular diseases such as Diabetic Retinopathy and AMD.

### Strategies for gene therapy

#### Gene augmentation

Gene augmentation or gene replacement is a very straightforward strategy for genetic recessive disorders caused by a dysfunctional gene, where a functional copy of a gene is delivered to affected cells in order to restore the expression of an inadequately functioning gene (85, 86). In this approach, there is no requirement to modify the native genomic DNA sequence of the affected cells as the augmented function is provided by supplementary DNA that coexists in the nucleus of the cell. The success of this strategy is determined by two factors (i) the inserted gene must produce physiological and/or sufficient levels of the normal protein (ii) the disease effects are still in a reversible state (iii) In case of IRDs, the disease is recessive or X-linked. This method is one of the most widely used strategies in gene therapy trials for ocular diseases including the FDA-approved Luxturna. Three fourth of patients with achromatopsia carry mutations in either *CNGA3* or *CNGB3* genes that encode the cyclic nucleotide-gated channel in cone (87). There are studies that have shown that through gene augmentation using AAV (Adeno-associated virus) vectors carrying either of the two genes, improvement has been observed in murine, ovine and canine models (88–90). Intravitreal injection of an AAV8 vector carrying the Retinoschisis 1 gene (*RS1)* in a mouse model with X-linked retinoschisis, caused by a mutation in the *RS1* gene, exhibited significant improvement in retinal structure and function post-treatment and has moved to a phase 1/2 clinical trial (NCT02317887) (91). Gene augmentation strategy provides a long-term persistent expression of secreted therapeutic proteins to deal with non-genetic retinal disease, such as AMD. The interim results of Regenxbio from phase I, open-label dose increasing trial assessing the efficacy and safety of the subretinal injection of a novel AAV8 vector (RGX-314) encoding a soluble anti-VEGF monoclonal antibody fragment (NCT03066258) were encouraging (92). The expression levels of the protein were observable at 1 month in a dose-dependent manner, with sustained expression observed at 6 months in patients treated at a dose of 6e10 vector genome (vg) per eye. Over the period of 6 months, minimal or no anti-VEGF injections (50% of patients) were required by patients treated at that dose, with preservation of central retinal thickness and assessments of best-corrected visual acuity (BCVA) vs. baseline showed either conservation or improvements in visual acuity. Whether this durability can extend beyond 1 year still needs to be evaluated. An AAV2 vector carrying the *ND4* gene (GS010) for LHON patients showed good safety and efficacy results in a number of 1/2 trials and several phase three clinical trials are currently being done (93). These examples of tailored gene therapy approach for IRDs highlight the incredible potential for genomic precision medicine.

#### Gene editing

This approach, focuses directly on editing the genome by correcting or removing the mutant gene which is superior to gene augmentation particularly in autosomal dominant conditions as well as point mutations. While recombination and genome editing technologies such as ZFN (Zinc Finger Nucleases) and TALENs have been available for some time, the most specific and advanced genome editing technology to date is Clustered Regularly Interspaced Short Palindromic Repeats (CRISPR)–Cas editing system. CRISPR based editing depends on the associated Cas proteins, the cognate guide RNA which targets the nuclease to particular sites desired depending on available sequence contexts (94). Even though gene editing has certain advantages over gene augmentation, it does carry a risk of inducing off-target mutations caused by the nucleases. Clinically, CRISPR-Cas9 editing method has been developed for treating *CEP290* mutation associated with autosomal recessive LCA10, IVS26 c.2991 + 1655A > G, p.Cys998X (95). This mutation causes the addition of an ambiguous exon in the final gene product that has a premature stop codon. The approach to remove the aberrant splice-donor is in a current Phase I/II trial led by Editas Medicine/Allergan (NCT03872479), marking the first *in vivo* human use of CRISPR-Cas9 technology (96, 97). The first *in vivo* gene editing clinical trial for LCA10 patients (NCT03872479), assessing the safety, tolerability, and efficacy of AGN-151,587 (EDIT-101, Allergan, Dublin, Ireland), a CRISPR-Cas system was commenced in March 2020 (98). This multicentre trial is a landmark in gene therapy being the first to directly administer gene-editing therapy via subretinal injection.

#### mRNA-based gene therapy

Suppressing the faulty gene to restore normal function is another mechanism of specific gene-targeted therapy, particularly in autosomal dominant diseases, infectious conditions and in age-related disorders. The most successful mechanism in this context is the use of antisense oligonucleotides (AONs) which are short synthetic single-stranded RNA or DNA that bind to the complementary mRNA and brings about its multiple effects that can either hamper or correct target gene expression or alter the pre-mRNA splicing causing splice site inclusion or exclusion (99). Importantly, the first AON approved by FDA for marketing was for the treatment of cytomegalovirus retinitis: fomivirsen (Vitravene). Preclinically developed AONs can be used to treat diseases caused by deep intronic mutations resulting in the insertion of pseudo-exons with premature stop codons (100), such as in choroideremia (101); abnormal splice transcripts that cause exon skipping, as in Stargardt disease (102, 103); and to repress the gain-of-function Pro23His mutation in autosomal dominant *RHO*-associated RP, which recently entered a Phase I/II clinical trial (QR- 1123; ProQR Therapeutics). QR-110 (sepofarsen), an antisense oligonucleotide is being tested to restore accurate splicing in patients with LCA10 having a point mutation in the ciliopathy gene that encodes centrosomal protein 290 (CEP290) (104). The treatment was conducted via intravitreal injection in one eye every 3 months and was done for four doses and assessed functionally over 1 year. Results for this trial were recently described by press release, with improvements in full-field light sensitivity threshold (FST), best-corrected visual acuity (BCVA), and mobility. Visual acuity improved at 3 months with significant differences between treated and non-treated eyes, leading to Phase II/III trials in 2019. Also, presently one of the most prevalent (>30%) *USH2A* mutation c.2299delG, which leads to a frameshift and truncation of exon 13 is being targeted by designing AON to exclude exon 13 which is currently in Phase Ib/II trials (QR-421a) (105).

The discovery of the RNAi pathways led to the use of shRNA to inhibit the faulty gene expression in ocular diseases. This gene silencing strategy would be suitable, for some dominant genetic diseases, ocular cancers, or certain infectious diseases. This strategy introduces an RNAi expression cassette that either inhibits the mutant gene or interferes with the mutant protein activity. Inspiring safety results have been reported with siRNAs targeting the *RT801* gene or caspase-2 (QPI-1007) in glaucoma, in non-arteritic anterior ischemic optic neuropathy (NAION), etc (106). There are success stories of gene silencing for several diseases in preclinical studies and currently, some are progressing toward clinical trials.

All the different strategies of gene therapy mentioned above have certain limitations. With gene augmentation therapy, safety is a concern as there is a chance of insertional mutagenesis in the host genome in case of integrating vectors and waning of expression from non-integrating vectors over time leading to loss of therapeutic effects. Despite success, gene silencing strategies are typically limited by incomplete suppression of the mutant protein. For both gene editing and RNAi mechanisms, the major concerns like off-target effects and extended toxicity of the enzymes need to be considered. The potential off-target effects of these techniques need to be considered carefully and followed diligently in human studies. Overall, further studies in the safety and efficacy profile of these new modalities are critical.

#### Mutation independent gene therapy strategies

One of the major causes of blindness is IRD, caused by a variety of mutations in more than 300 genes (3). Vision loss in all IRDs regardless of the relevant mutation is the final outcome which is typically due to the death of photoreceptor cells. While every retinal disease has its own genetic mutation, the common disease physiology, i.e., the loss of photoreceptors, is not always addressed sufficiently by the current approaches. As noted in the gene therapy clinical trials of LCA2, the visual function reduced after a few years even after rescuing the primary gene mutation in the retina. The reduction in the visual function was due to the persistent death of photoreceptors that diminished the efficiency of AAV.RPE-65 gene therapy (107–109). Photoreceptor death is a cumulative consequence of photic damage, aberrant cell signaling, endoplasmic reticulum stress, mitochondrial dysfunction and chronic inflammation (110). It was noticed that in animal models lacking *RPE656*, *ABCA4*, etc., loss of photoreceptors through apoptosis occurred spontaneously in response to light damage (111–113). The IRDs are being treated currently through a gene therapy approach based on mutations specific to a small subset of patients who carry the exact cognate gene mutations (114).

Both apoptotic and necrotic pathways are responsible for the death of the photoreceptors in all IRD (115, 116). These mechanisms of cell death commonly converge on the executioner Caspase 3 which gets activated by both intrinsic and extrinsic programed cell death pathways (117). Therefore, inhibiting apoptosis through anti-apoptotic proteins is important when regulating cell death processes (118, 119). Further, the functioning of retinal cells is dependent on a variety of chaperones that are essentially required for homeostatic functions in the cells (120). Moreover, animal studies have revealed that neurotrophic factors could inhibit cell death and escalate functional capacity in the retina, optic nerve, and brain (121–126). The potential neuroprotective effects of neurotrophic factors, including brain-derived neurotrophic factor (BDNF), ciliary neurotrophic factor (CNTF), glial cell-line derived neurotrophic factor (GDNF), and nerve growth factor (NGF), makes them efficient therapeutic candidates for neurodegenerative diseases (121). Glaucoma is a neurodegenerative disease of the eye that is characterized by damage to the optic nerve, particularly due to high intraocular pressure (IOP), and continuous degeneration of retinal neurons called retinal ganglion cells (RGCs) (127, 128). Presently, reduction of IOP is the main focus for the therapy of glaucoma, but neuroprotection may also be beneficial. BDNF is a potential neuroprotective agent especially for RGCs. It has been observed that RGCs can be protected from damage by exogenous application of BDNF to the retina and by increasing BDNF expression in retinal neurons using viral vector systems (129). Moreover, inducing BDNF expression by agents such as valproic acid has also been advantageous in elevating RGC survival (129). NGF has also been implicated in retinal damage regression. It has been reported that NGF administration exerts a rescue effect on photoreceptors *in vivo* (122). Therefore, focusing on these three strategies to select targeted genes that need to be augmented to avoid photoreceptor death can have therapeutic potential.

**FIGURE 2:**
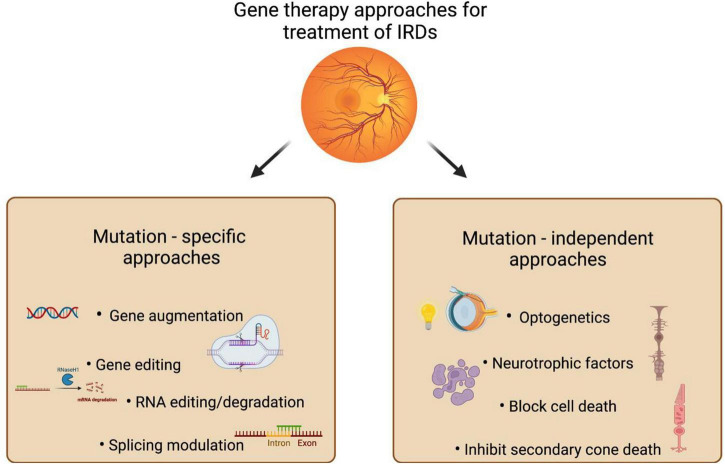
Gene therapy strategies for IRDs. This schematic represents the potential therapeutic approaches that gene therapy offers for various retinal diseases. Gene/mutation-based approaches are preferred when the knowledge of the genetic cause of the disease are known. Mutation-independent approaches act on common pathways that underly retinal damage and help in treating a large fraction of patients with genetically heterogeneous and complex retinal diseases.

An important challenge in IRD therapy is the development of broad application therapies, which are independent of gene mutation and act on common pathways that underly retinal damage (Figure 2). One such mutation-independent approach that is ideal for treating diseases with initial photoreceptor degeneration is activation of neuroprotective pathways. Expression of the neurotrophic factors using AAV mediated delivery can enable stable transgene expression and therapeutic efficacy. Studies have shown the ability to prevent photoreceptor death in several mouse models of retinal degeneration by using neurotrophic factors such as CNTF, BDNF and pigment epithelium-derived factor (PEDF) (130–132). In a more recent study, a single AAV vector expressing both BDNF and its receptor, the tropomyosin-related receptor kinase-B (TrkB), showed significant long-term RGCs survival and improved positive scotopic threshold responses in a model of optic nerve crush and in a model of high-tension glaucoma (HTG) (133).

Another broad approach is optogenetic therapy, which aims to restore vision in late-stage IRDs. Here the vision is restored by using pre-existing retinal neural synapses. It targets genes such as opsin genes that encode photosensitive proteins to selected retinal cell types, thereby converting them into replacement photoreceptors (134, 135). In this approach, opsin genes are inserted into a gene expression cassette and delivered via an adeno-associated viral (AAV) vector into neurons *in vivo*, which leads to the transduced neurons being rendered photosensitive. Intravitreal injection with an AAV containing an optogenetic expression cassette in a patient with late-stage RP showed that the treated eye gained the ability to perceive, locate, and count various objects whilst using the light stimulating goggles (136). Another study showed improvements in the visual function in two rod-cone dystrophy (RCD) mouse models with mutations in two different genes, after treatment with AAV mediated expression of G-protein coupled inwardly rectifying K (GIRK) channel. Furthermore, they observed the expression of cone opsin and cone arrestin in cones of late-stage rod-cone dystrophies (RCD) patients, validating the use of GIRK-mediated gene therapy in humans (137). Currently, there are several clinical and pre-clinical trials using different types of optogenetic molecules expressed alone or in combination targeting different cell populations (138). These studies further indicate the potential of mutation-independent approaches in treating a large fraction of patients with genetically heterogeneous and complex retinal diseases.

### Viral vectors for ocular gene therapy

Viruses are the most common gene therapy vector taking into consideration their capability to infect and release their genetic content into a target cell through the process of transduction. High expressivity, long stability, transgene carrying capacity, low immunogenicity, and low risk of mutagenicity are the characteristics desirable of a gene delivery vector (139). In gene therapy, retroviruses, and adenoviruses were among the first used vectors due to their high levels of infectivity and large carrying capacity. However, the risk of insertional mutagenesis in case of lenti/retroviruses and the strong immunogenicity and short duration of expression in case of adenoviruses has limited their use for human gene therapy in IRDs (140, 141). AAV is presently the most commonly used vector for retinal gene delivery. Compared to other vectors, AAV exhibits low immunogenic response, non-integrating nature and low retinal toxicity (6, 142). Particularly, recombinant AAV genomes remain as episomal concatemers in transduced cells causing extended expression of the transgene in non-dividing retinal cells. When administered subretinally, AAV vectors can efficiently transduce RPE and photoreceptor cells.

Till date, there are 13 known serotypes of AAV (AAV1-AAV13), which differ from each other in their capsid protein sequences and ability to bind to different surface receptors/co-receptors on target cells that helps define their relative tropism (143). AAV2 is the prototype serotype and one of the first to be tried for human retinal applications. Since AAV2 is efficient in gene delivery to the RPE, it has been widely used in clinical trials (144). In 2001, success of AAV mediated therapy of *RPE65*-/- dogs paved the path for several phase I and II human clinical studies. Spark Therapeutics (NCT00516477), University of Pennsylvania/National Eye Institute (NCT00481546), University College London/Targeted Genetics (NCT00643747), and Applied Genetic Technologies (AGTC)/Oregon Health and Science University/University of Massachusetts (NCT00749957) have conducted phase I/II trials using AAV2 with different vector designs, vector volumes, and administration procedures. All these clinical trials indicated safe delivery of AAV2 to the retina despite of the differences in clinical trial conditions. Voretigene neparvovec-rzyl (Luxturna), an AAV2 vector carrying the *RPE65* gene, marked the first commercially available gene therapy after its FDA approval in December 2017. It is used for *RPE65* mutation-based retinal dystrophies, namely, LCA2 and a subgroup of autosomal recessive RP. Luxturna is given to patients with viable retinal cells through subretinal injection and hence patients having more advanced forms of the disease will not be allowed for treatment. After successful studies of AAV-mediated delivery of *RPE65* in a canine model, multiple independent groups showed the safety and efficacy of various AAV vectors in phase 1 and 2 clinical trials (Table 1).

Preclinical studies have shown that pseudotyped AAV2 ITR transgenes in other serotypes like AAV5 and AAV8 can increase transduction levels in retinal cell types (145–148). These pseudotyped vectors are presently being used in trials for a few ocular diseases like autosomal recessive and X-linked recessive RP and LCA. In addition, various mutations have been introduced into the native AAV capsids across different serotypes that have resulted in further enhanced viral transduction efficacies (ref—Arun Srivastava reviews). For example, mutating tyrosine (Y) to phenylalanine (F) on the capsid surface of wildtype AAV increases transduction efficiency (149–151). AAV2tYF is an example of one such vector with triple Y-F mutations that are being tested in multiple IRD clinical trials like X-linked RP (NCT03316560), achromatopsia (ClinicalTrials.gov Identifier: NCT02599922, NCT02935517), and XLRS (NCT02416622). RP caused by *PDE6B* mutations has autosomal recessive transmission, and a phase 1/2 trial of an AAV2/5 vector carrying this gene is ongoing.

The small size of AAV with a diameter of 25 nm allows stronger diffusion through the layers of cell and extracellular matrix but limits its delivering capacity of transgene cassette to 4.7 kb of DNA highlighting the need for AAVs that can package larger genes (152). Dual AAV vector strategies are being used where a transgene larger than the ∼4.7 Kb is separated and packaged into two AAV vectors for later reconstitution within the target cell (153). Multiple different dual vector strategies including fragmented, trans-splicing hybrid, and overlapping have been tested (154–157). Stargardt disease and Usher syndrome (USH) are two IRDs caused by mutations in large genes that surpass the capacity of AAV. The use of AAV dual vector strategy has been used to target *MYO7A* for USH in several studies, but this still needs to be translated to clinical trials, but with Stargardt disease, inspiring results have been obtained that has the possibility of better treatments in the future based on AAV vectors (158, 159). Recently, triple AAV vectors have been used to enhance gene transfer capacities up to 14 kb for USH1D (*CDH23* mutation) and Alström Syndrome type 1 (*ALMS1* mutation) where gene sizes are too large for dual vectors (160). The limitation of triple and dual vectors is its lower photoreceptor transduction efficiencies with the current generation of vectors. As an alternative to AAV dual vectors, vectors with larger capacities like lentiviruses may be employed in such specific cases. Lentiviruses have a larger transgene carrying capacity; the equine infectious anaemia virus (EIAV) has a transgene packaging capacity of 8 kb (161). Forty Clinically important genes that are larger in size like *ABCA4* and *MYO7A* for Stargardt disease and USH1B, respectively, can be packaged into EIAV (162). EIAV has displayed good efficacy as a vector in animal models of both diseases and has made its way to clinical trials. As lentiviruses can integrate into the genome of the cell, there is always a risk of insertional mutagenesis that can lead to cancer (161).

### Non-viral vectors for ocular gene therapy

Compared to viral vectors, non-viral vectors are non-pathogenic, less immunogenic, have a lower risk of insertional mutagenesis, have the potential for repeated administration, and can be easily produced on a large scale. They can be engineered for a larger cargo capacity; however, they are generally less efficacious in transgene delivery and less durable in sustained gene expression compared to viral vectors. Often, the cargo DNA is complexed with other chemical molecules or forced to enter the cells and nucleus through physical procedures. Emerging non-viral technologies through chemical methods include synthetic polymers (163) and nanoparticles (NP) (164), physical particles, lipid-based delivery systems (165, 166), and functionalized cell-penetrating peptides (162).

DNA NPs have not been assessed in gene therapy clinical trials of ocular diseases, due to low or short-term transduction (167). Polymers, liposomes, peptides compacted DNA are examples of NPs that have been tested as gene delivery systems for retinal diseases (167, 168). The low cost of manufacturing, the convenience of transferring large vectors without any immune reaction, and the ease to manipulate its chemical property to suit DNA delivery are the advantages of using NPs (169). The latest achievement in this field is the DNA NP developed by Copernicus Therapeutics that includes a single plasmid DNA packed with a 10-kDa polyethylene glycol (PEG)-substituted 30-mer lysine peptide (CK30PEG), which reaches the nucleus faster through the process of nucleolin-dependent endocytosis (170). These compacted DNA NPs after being delivered in the subretinal space, target the photoreceptors and RPE cells without remarkable toxicity, with a stable expression up to 2 years in mice (171, 172). CK30PEG NPs have been evaluated in preclinical trials for RP, LCA, and Stargardt disease in mouse and rabbit models (169). It was observed that even when delivered by intravitreal injection in non-human primates, NPs were able to transduce retina and RPE (173). With a large carrying capacity, DNA NPs have an advantage over AAV vectors having a small carrying capacity for ocular gene replacement therapy, on the condition that long-lasting expression is proven in large animals.

Apart from chemical methods, physical methods of non-viral vector methods have evolved to enhance cellular entry of DNA into ocular cells, examples are iontophoresis (174, 175), bioballistic (176), electrotransfection (177), magnetofection (178), optoporation (179), and sonoporation (180). Among these, DNA electrotransfection also known as electroporation or electropermeabilization is the most promising method for ocular gene delivery (162). This method is based on administering an electric field to the cell to create pores in the cell membrane, facilitating the penetration of naked plasmid DNA, and assisting its cellular uptake through electrophoresis (181). Electrotransfection after subretinal injection of naked plasmid resulted in effective transduction of the neuroretina and RPE in newborn rodents and adult animals, respectively (182–184). It was observed that injecting a soluble VEGF receptor-1 (sFlt-1)-encoding plasmid into the suprachoroidal space followed by electrotransfection led to the transduction of choroidal, RPE cells, and potentially photoreceptors causing a remarkable reduction of laser-induced choroidal neovascularization (185). Increased photoreceptor survival was obtained using BDNF gene transfection in RPE on performing subretinal injection and electroporation in the Royal College of Surgeons (RCS) rat model (162). Intravitreal injection of DNA followed by electroporation caused transfection of adult rat RGCs (186). Electroporation of DNA plasmids into ciliary muscles can present as a bio-factory for therapeutic proteins (187, 188) and has proven efficient in animal models of uveitis, RP, and wet AMD for up to 6 months. This program is undergoing clinical trials for non-infectious uveitis (ClinicalTrials.gov NCT03308045) (162). Even after successful preclinical trials, this method has disadvantages due to the need for invasive surgery to place microelectrodes for exhibiting an electric field in the vicinity to targeted cells making the translation of this method to humans challenging.

### Promoter Specificity for Personalized Therapeutics

The need for an effective promoter to pilot high and clinically relevant levels of therapeutic gene expression is important. The selection of promoter should be such so that a convenient dose of the vector would be enough for treatment which will help to overcome the immune responses or cellular toxicity resulting from multiple or high virus dosage. To avoid unwanted transgene expression at off-target areas and to ensure cell type-specific gene expression, the use of a gene-specific or cell-specific promoter is an utmost requirement. Reduction of the cone outer segment, shortening of the outer nuclear layer, and dysmorphic pigment epithelium are some of the features of retinal toxicity for which promoter selection requires careful evaluation. Over the past few decades, broad expression promoters have been widely used in gene therapy studies such as CMV cytomegalovirus (CMV) (189), chicken beta-actin promoter (CAG) (190), and human ubiquitin C promoter (UbiC) (191, 192). A small number of promoters specific to the retina are being used like RPE-specific promoter, *Best1* (bestrophin-1), and *RPE65* promoter (193, 194). There may be a requirement for a more strongly regulated control of protein expression level to avoid toxic build-up in the case of photoreceptor-specific genes. This demand paved the path for the development of a variety of custom promoters, in an effort to target expression in particular retinal cell types of interest as well as to provide physiologic expression of the exogenous transgenes. For example, the 1.7-kb human *L*-opsin PR1.7 promoter for cone-specific expression of *CNGA3* or *CNGB3* used in achromatopsia trials (195), photoreceptor-specific promoters such as human red opsin (*RedO*), human rhodopsin (*Rho*), human rhodopsin kinase (*RK*), mouse cone arrestin (*CAR*) (196), and human G-protein-coupled receptor protein kinase 1 (*hGRK1*) promoter that is being used for regular expression of exogenous *RPGR* in clinical trials for *RPGR* associated X-linked RP (197). Broadly active promoters generally might have increased expression levels compared to tissue-specific promoters. A study that was carried out comparing the transgene expression by five different promoters—cytomegalovirus immediate-early gene promoter (CMV), human alpha-myosin heavy chain (α-MHC), human desmin (Des), rat myosin light chain 2 (MLC-2), and human cardiac troponin C (cTnC) to drive LacZ mediated by AAV9 intravascular delivery in mice showed CMV overtopped all the other tissue-specific promoters by causing the highest level of transgene expression (198). At later stages of retinal degeneration, when maximum photoreceptor cells are lost, bipolar cells make a promising target for gene therapy. In the retina, bipolar cells are the first interneurons that receive direct input from the photoreceptors. They are divided into ON- and OFF-type bipolar cells that react to either light augmentation or light reduction, respectively, setting up the foundation for contrast vision. Even after obtaining success in targeting ON-bipolar cells (OBCs) in the mouse retina, they have remained inaccessible to human gene therapy due to the lack of a strong cell-specific promoter capable to direct an effective transgene expression in human OBCs. Recently, a group has described the design and functional assessment of 770En_454P(hGRM6), a human*GRM6* gene-derived, a small promoter that drives robust and highly specific expression in both the rod- and cone-type ON-bipolar cells of the human retina (199). Since the cone-dominated macula mediates high-acuity vision and is the primary target of gene therapies, expression in cone-type ON-bipolar cells is also of importance. In the rd1 mouse model of late retinal degeneration, 770En_454P(hGRM6)-driven middle-wave opsin expression in ON-bipolar cells attained lasting restoration of high visual acuity. Retina-specific promoters can enable precise manipulation of the inner retinal network and can pave the way for the clinical application of gene therapies for strong-resolution optogenetic vision restoration.

## Patient selection and patient stratification for personalized therapeutics

The concept of tailored therapeutics makes use of a multitude of testing options to precisely pinpoint management needs of individual groups of patients. In order for strategies developed from this approach to most likely benefit the patients, it is important to select and stratify patients into a more homogenous subpopulation by considering the common biological and molecular basis of disease (Committee on the Framework for Developing a New Taxonomy of Disease, 2011). It seeks to dichotomize patient population by the response of a patient to a specific type of treatment. Therefore, it is important to link a molecular profiling data to disease associated phenotypic abnormalities to identify the individuals likely to benefit from a new therapy. This in turn will also have a considerable economic impact as the drug development cost will be reduced and the risk of treating non-responders will be minimized.

### Family history and inheritance pattern

For clinicians to stratify clinical risk and assemble the correct multidisciplinary team and advise on possible treatment options that may benefit the patient, it is important to establish a correct and precise molecular diagnosis. To determine the etiology for any ocular disorder, it is important to have a clinical history and examination of the patient to guide which genetic test is most suitable to ascertain the cause of the suspected disorder. Having information regarding their detail birth history, pregnancy/family history, consanguinity along with details of disease features involving onset, progression, severity of disease is an extremely crucial element of the process. Usually, information obtained from family history can help determine the mode of inheritance of Mendelian disease such as autosomal recessive, autosomal dominant and X-linked. Of the 300 gene that have been associated to IRDs, approximately 70% are inherited in an autosomal recessive manner and 25% are autosomal dominant, with the remaining being either X-linked or mitochondrial diseases (3).

#### Autosomal Recessive Disease

Autosomal recessive disorder occurs due to the presence of biallelic variants on an autosomal chromosome. These variants can be CNVs, point mutations or even structural changes within a gene. The parents of the affected individual are carriers and are usually clinically unaffected or they could be affected with the same condition themselves. Usually, the risk of an affected patient’s child inheriting the autosomal recessive condition is small but very likely also depends on the population frequency of that specific pathogenic variant. In general, the risk of autosomal recessive conditions is much higher with consanguinity.

In inherited eye disorders, there are some common pathogenic autosomal recessive genes. For example, Stargardt disease is predominantly caused by biallelic variants in *ABCA4* gene. This determines the onset as well as the severity of the disease (200, 201). Some disease-causing variants in the same genes can be associated with different disorders. For example, biallelic variants in *USH2A* can be associated with syndromic disorder such as type II USH or non-syndromic RP (202–204). Many of the monogenic recessive disease could be treated with vector-based gene replacement approach as mentioned previously, where the cDNA of the mutated gene is delivered to compensate for the lack of protein production. About 5–10% of LCA cases is caused due to mutations in the gene encoding the RPE-specific protein RPE65 (141, 205). Amongst these, type 2 LCA turned out to be an excellent candidate as it was associated with a slowly progressing phenotype where the photoreceptors persisted over decades leading to a rather large window of opportunity for therapeutic intervention (206). AAV-mediated *RPE65* expression slowed down or reversed vision loss in both small and large animals, paving the way toward first application in humans (207, 208). Gene replacement therapy has also been implemented for other autosomal recessive retinal degenerative diseases including choroideremia, other forms of LCA, achromatopsia as discussed in detail under section Strategies for Gene Therapy. Other viral vectors such as lentiviral vectors have been used for some autosomal recessive disorder to deliver cDNA copies of the mutant genes that are too large to be carried by AAV, e.g., *ABCA4* gene associated with Stargardt disease and *MYO7A* associated with Usher’s syndrome (Table 1). However, one of the biggest concerns with this strategy is that the efficacy in these diseases might be limited by the tropism of lentiviral vectors.

Alternatively, gene editing strategies have also been used for treating recessive autosomal disorders such as recessive LCA10, which is caused by an intronic mutation in *CEP290* gene generating a novel splice donor site. Recently, a double sgRNAs combined with SaCas9 approach was reported to delete this intronic region in pre-clinical studies paving the way to clinical application (97). The safety and feasibility of this approach was showed in a pre-clinical work in non-immunosuppressed macaques using AAV5 vector with only mild inflammation.

#### Autosomal dominant disease

Autosomal dominant inheritance occurs due to a single heterozygous variant affecting one allele of an autosomal gene. Similar to autosomal recessive inheritance, these variants can be CNVs, point mutations, or structural changes within a gene. Affected individuals have a 50% risk for passing the mutated allele in each pregnancy to their child. In an autosomal dominant disorder, history of the unaffected parent or consanguinity of parents is of no relevance in determining the inheritance risks.

One of the most common pathogenic autosomal dominant genes seen in inherited eye disorders is *RHO*. It is mutated in approximately 30% of autosomal dominant RP cases (209, 210). In autosomal dominant optic atrophy, which can be associated with extra-ocular features, *OAP1* variants accounts for approximately 65–70 of the cases (211, 212). Aniridia, leading to a variable degree of iris and foveal hypoplasia, nystagmus, cataract, glaucoma and corneal keratopathy usually affects 1:40,000–100,000 births and is caused by *PAX6* variants (213).

In general, treating autosomal dominant retinal diseases is a more formidable challenge unlike autosomal recessive retinal conditions, because researchers need to deal with one gene copy expressing a toxic protein and another that’s functioning normally. To add to this complexity, autosomal dominant diseases can be caused by dominant-negative or gain-of-function mutations. In case of dominant-negative mutations, the encoded protein has an antagonistic effect to the wild-type one and in gain-of-function mutations, it can have a new function, also leading to toxicity. Therefore, for such mutations, along with healthy gene supplementation, the mutated gene needs to be silenced to inactivate the detrimental effect (214). Gene correction could also serve as an alternative gene therapy option in such cases.

Among the variety of *RHO* mutations that accounts for autosomal dominant RP, P23H, and P347L are the two most prevalent mutations (215, 216). P23H mutation has both dominant-negative and gain-of-function effects, with protein retention in the endoplasmic reticulum (217). Allele-specific disruption has been developed to treat this disease, where the main target of genetic silencing strategies is the mRNA transcript. The function of the mRNA transcript is inhibited by antisense RNA-based, ribozyme-based and more recently by small interfering (si)RNA-based and micro (mi)RNA-based, approaches. Since *RHO* is an essential gene for the retinal function, its complete suppression will induce pathological phenotypic. To prevent this, supplementation by the addition of exogenous rhodopsin is required to achieve optimal therapeutic benefit. Therefore, more efforts have been focused on coupling the silencing and the replacement. Amongst all the many studies that are in progress to find a successful therapy for autosomal dominant RP, especially for *RHO* mutations, the ‘silence and replace’ strategies seem to be the most promising. Additionally, a mutation independent silencing seems to have better potential clinical applicability especially for autosomal dominant RP as that could be applied to all the different mutations and therefore would be useful to more patients.

Another approach to treat dominant autosomal diseases is gene editing using CRISPR Cas9 which is still in pre-clinical stages due to more complex issues surrounding dominant negative mutations. Complexity of this approach also arises from the need to specifically target the mutant allele by using mutation specific sgRNA or Cas9 variants with PAM sequence including the mutation. Several groups have successfully applied this strategy *in vivo* in RHO.P23H mice. Specific disruption of the mutated gene was achieved by using sgRNA specific to dominant rhodopsin mutation combined with Cas9 VQR variant (215, 218). However, in the bigger picture need for such specificity makes this a costly mutation dependent strategy, thereby reducing the number of patients that can benefit from such treatment. Additionally, as this strategy does not compensate for the inactivation of the mutant allele, it may lead to haploinsufficiency.

#### X-linked disease

X-linked inheritance occurs because of a variant affecting a gene on the X-chromosome. Men are primarily affected through the hemizygous pathogenic mutation, and female carriers could be asymptomatic, mildly symptomatic or display manifest signs of disease, such as seen in X-linked RP (219). Due to the phenomenon of X-inactivation, women could be clinical affected at varying degree based on what proportion of healthy X chromosomes are inactivated in a carrier state.

In an X-linked recessive disorder a female carrier has a 50% risk of passing the pathogenic mutation to her progeny. Choroideremia is one of the most common X-linked recessive disorders in IRD and is caused by mutations in *CHM* gene. It is a chorioretinal dystrophy characterized by progressive degeneration of the photoreceptors, RPE and choroid (220). X-linked RP represents 8% of RP and is caused mostly by variants in *RPGR* gene (221, 222). Another X-linked recessive disease is Lenz microphthalmia syndrome caused due to *BCOR* variants and is characterized by cataracts and microphthalmia (223).

A female’s healthy X chromosome does not compensate in an X-linked dominant case, unlike what we see with X-linked recessive inheritance. Therefore, females and males can both be affected. However, X-linked dominant diseases usually affect the males more severely than heterozygous females and many such conditions are lethal in males during early life. Each child of a female patient with an X-linked dominant disorder has a 50% risk of being affected. X-linked dominant disorders are very rare. One example is incontinentia pigmenti which usually only affects females and is caused by pathogenic changes in the *IKBKG* gene (224).

### Clinical and genetic inclusion and exclusion criteria

Establishing inclusion and exclusion criteria is an important and required practice when designing high-quality treatment protocols. Inclusion criteria include the key features of the disease that will be help form the treatment protocol such as type of mutation causing the disease, age, onset of disease, progression stage of the disease, and other genetic and clinical features related to that disease. In contrast, exclusion criteria involve individuals who present with additional characteristics that could interfere with the success of the treatment or increase their risk for an unfavorable outcome even if they meet the inclusion criteria. Usually, exclusion criteria include eligible individuals in whom the stage of their retinal degeneration precludes them from the therapeutic window or have comorbidities that could either bias the results of the study or have pre-existing immune activity against the vectors/drug which may increase their risk for adverse events. It is critical to establish detailed clinical history and prior treatments such that contra-indicative drugs and co-morbid pathologies may be avoided, particularly in case of retinal diseases associated with syndromes or systemic conditions.

#### Therapeutic window

While progress is being made for retinal gene therapy, it is important to acknowledge that the key limiting factor for gene therapy application in IRD patients is the disease progression status and stage of photoreceptor degeneration. A key challenge of retinal gene therapy is for the therapy to be done prior to complete loss of the target cells, usually the photoreceptors or the RPE. Early detection, intervention, and physiological alteration to slow or stop the loss of photoreceptors cells remains an important factor limiting the therapeutic window for vision restorative gene therapy. Studies have shown that the potency and efficacy of gene transfer to the retina at late stages of the disease is less robust. In *REP65* associated LCA retinal gene therapy trials, the greatest improvement was observed in the younger children (225, 226). One of the major challenges with such disorders is that in several cases the patients may not even develop severe symptoms until much later, at which point the therapeutic target cells are atrophied. Another aspect is that the threshold for many of the progressively degenerative diseases is not defined, as degeneration cannot be stemmed in a cell autonomous fashion. For identifying the appropriate therapeutic approach for each patient, elucidation and diagnosis of that transition point plays an extremely essential role.

Some progressive diseases have a large window of therapeutic opportunity where interventions can be made prior to the loss of retinal cells, such as, achromatopsia, where cones stay anatomically intact. Another example that provides a large therapeutic window is congenital stationary night blindness where there is no degeneration but a functional loss at the bipolar cell level (227, 228). On the other hand, in LHON, within the first year of the onset of the disease, the affected RGCs causes progressive loss of vision, thereby severely restricting its therapeutic window (229). In general, gene replacement or correction for most cases of RCD is uncertain to provide a life-long benefit once photoreceptors have started to degenerate. In case of many unknown and dominant mutations where gene replacement cannot be used as the treatment strategy, the secretion of survival factors delivered in form of gene therapy in combination with gene replacement may be beneficial strategy (230). Metabolic issues, inflammation and oxidative stress that arise in cones secondary to the loss of rods can be combated using several varieties of survival-enhancing factors (231). Ectopic expression of microbial opsins (optogenetics) has shown to help with restoration of light sensitivity in cone cells even after the loss of its outer segment (232). Such approaches give further opportunities to salvage vision via the use of gene therapy, especially in advanced ocular diseases.

#### Genotype-phenotype correlations

Usually, retinal diseases are diagnosed on the basis of family history, retinal fundus features, standard tests of peripheral vision and visual acuity, and psychophysical measurements (233–235). Recently, with the advancement in molecular genetic testing, specialists in retinal degeneration have come up with gene-based diagnostics (207) which have evolved from detailed knowledge of genotype-phenotype correlations. Yet, the detailed knowledge of molecular mechanisms and understanding of the natural history of many retinal diseases remains to be established. Several conditions that we thought to be symmetrical previously are now found to have asymmetries which further point to the variegation of local cellular expression patterns. Therefore, most studies now represent cohort data that includes information regarding the correlation of clinical features and genetic alterations. For example, in Choroideremia, both eyes usually undergo degeneration in a similar way where central macular function stays well beyond the loss of the peripheral retina. However, there is a difference in the locations of the transition zones and the exact areas occupied by the remaining cells between the eyes (220, 236, 237). Patients or even siblings with mutations in same genes often demonstrate differences in disease penetration and progression. As more research and clinical studies on genotypes are described across many ethnicities with IRDs, the overlap between clinical features as well as genes mutated is evolving. In another example, exome panel based molecular diagnosis of IRD and was successful in identifying 124 mutations, with 79 novel mutations, in a cohort of 179 Chinese patients (238). They unraveled new genotype-phenotype correlations of IRD and recognized a novel candidate gene for non-syndromic RP. Another study involving 14 families of Northern Pakistan described the genotype-phenotype correlations of LCA revealing six novel, homozygous mutations in genes *AIPL1, LCA5* (3 families each), *RPGRIP1* (four families), *RPE65*, *CRB1*, *TULP1* (one family each) and linkage to the *LCA9* locus. The study demonstrated the differences in clinical phenotypes, for both the anterior and posterior segments observed between patients with different or identical mutations in the LCA genes and also suggested that at least some of the phenotypic variations are age-dependent (239). Genotypes in RP are heterogeneous as a patient with the same mutation may exhibit different phenotypes (240, 241). RP is inherited as autosomal dominant/recessive or as X-linked (XLRP). Mutations in *RPGR* and RP2 caused XLRP in 8.5% of probands (212). It has been reported that females who were affected with RP retained better visual functions compared to males (242, 243). A novel c.350G > A sequence in exon 5 of *RPGR* was identified on DNA analysis that segregated with disease in families (243) providing a specific example of phenotype associated across subjects linked genetically. In another example, a Swiss family of five generations affected with dominantly inherited RP caused by a rare T494M mutation in precursor mRNA processing factor 3 (PRPF31) was identified to relate phenotype to the particular mutation (244). This report was based on the large pedigree and gave a better understanding of phenotype-genotype explanation as caused by *PRPF31* mutation. These examples reinforce that natural history studies are critical for the success of gene therapies. They provide the investigators with information regarding the optimal stages of intervention and outcome measures for therapies.

## Application of human pluripotent stem cells as ocular regenerative medicine

Due to their self-renewal capacity and ability to differentiate into multiple cell lineages, embryonic stem cells (ESC) and induced human pluripotent stem cells (iPSC) are currently under investigation for the treatment of age-related macular degeneration and other retinal disorders (245, 246). Till date, no stem cell-based therapy for retinal disease has been approved by the U.S. Food and Drug Administration, however there are multiple candidates in development. In the eye, iPSCs have the capability to regenerate or replace tissue, such as RGCs in glaucoma, or RPE in retinitis pigmentosa or AMD-related geographic atrophy (GA) (246–248).

Transplantation of retinal pigment epithelium cells is a popular application of stem cell therapy in ophthalmology. It was in 2012, the first human studies of stem cell-based RPE transplants in AMD and Stargardt Disease were published (249). Previously, two prospective clinical trials of subretinal transplantation of hESC (human Embryonic Stem Cell)-derived RPE cells were performed in nine patients with Stargardt macular dystrophy and in another nine patients with atrophic AMD. After surgery which was combined with immunosuppression, 72 percent of patients displayed increased subretinal pigmentation at the site of the transplant, indicating the presence of the injected cells (250). There was no evidence of serious adverse outcomes in visual acuity, static perimetry, electroretinography visual field, or reading speed, and there was no evidence of acute rejection. After 4 years of follow up, none of the eyes showed abnormal growth like teratoma and no eyes developed proliferative vitreoretinopathy or a retinal detachment (250, 251). Similar results were obtained by Won Kyung Song, MD, of Korea’s Bundang Medical center, and co-workers (252).

The use of iPSC (induced Pluripotent Stem Cell)-derived RPE transplants in human trials is a more recent development compared to ESCs (Embryonic Stem Cells). In 2014, the first human trial using iPSC-derived RPE subretinal transplants was reported (253). The first person to receive an iPSC-derived therapy was a 70-year Japanese woman who didn’t receive immunosuppression, compared to ESC-derived RPE transplantation studies (250, 252). Importantly, the subject didn’t display any detrimental ocular effects on follow up after a year. The transplanted sheets were intact and there was stabilization of vision. Enrollment of additional subjects was suspended temporarily as mutations were observed in a second subject’s iPSCs, which weren’t detectable in the patient’s original fibroblasts. This study was resumed in 2016 with significant modifications (253).

Though stem cell-based therapies have potential, this field is still resolving critical issues such as incomplete differentiation, grafting efficiency and risk of teratoma formation. In the last several years, stem cell-based therapies have progressed from *in vitro* and animal models to human trials with limited efficacy data.

## Conclusion

**FIGURE 3:**
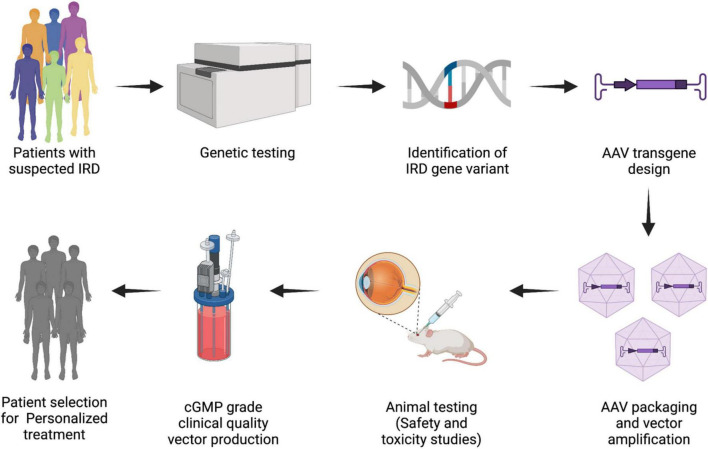
Overview of bench to bedside approach for personalized treatment. Advances in genomic analysis allow for genetic evaluation and diagnosis of IRDs. Further evaluation of the genetic etiology helps in translating it to gene therapy based personalized therapeutic options. This schematic focuses on AAV as the vectors of choice for gene therapy in the retina, due to the limitation of lentiviruses and adenoviruses in transducing the mature retina.

Precision medicine is a multi-dimensional approach for physicians to personalize therapy. Precision medicine identifies the growing relevance of patient-specific genetics and genomic studies in medical ophthalmology. Recent advances made in genomics, proteomics, gene therapy, and nanotechnology have increased the possibility to explore options for effective personalized therapeutics with minimum side effects. Abiding by the principles of precision medicine, ophthalmologists are required to procure patient genetic profile results and to combine this information with overall assessment of family history, lifestyle, environmental factors, eye health, clinical history, and ophthalmology examinations including retinal function tests. The main goal would be to identify key risk factors for disease, enable early-stage diagnosis and evaluate therapeutic modalities to improve, preserve, and restore vision. With the growing knowledge of genetic mechanisms in inherited eye diseases, it has become easier to provide personalized treatments (Figure 3).

Pathogenesis of retinal dystrophies have been associated with mutations in over 300 genes with more being discovered. For a long time, patient reassurance and counseling were the primary tools with clinicians for IRD patient management. The dramatic advancements of human gene-based therapeutics in the past couple of decades due to extensive knowledge and technological improvements, has now provided new options for patients, counselors and clinicians. After several years of exhaustive research, gene therapy has now entered a promising era of sustained research, indicated by the first ocular therapeutic gene product Luxturna approved by the FDA. Furthermore, two other disorders, Choroideremia and LHON, are currently in the Phase 3 trials (Table 1). Success of various gene therapy trials have increased expectations from the research and medical community, bringing hope of effective treatment for genetic diseases in the near future (2, 254, 255). With the sophistication of current molecular and surgical techniques, IRDs are a good target due to their well-defined and typically monogenic basis. Moreover, IRDs usually progress faster for regulatory approvals due to their orphan status and this can improve early patient access to treatment (256). Considering the diversity of mutations and the involvement of multiple genes, continued knowledge accrual and personalization of therapeutic strategies for optimal outcomes would be beneficial in IRDs. Gene therapy approaches along with genetic testing will continue to improve medicine where personalized treatment relevant for each individual will eventually define the standard of care. In future, a greater number of patients will gain access to precision care as economics of delivery and production of personalized drugs improve.

## Author contributions

ArG conceptualized the manuscript. PP and SR wrote the manuscript and made figures. AnG, ArG, and BP assisted in writing and helped with editing. All authors contributed to the article and approved the submitted version.

## Conflict of interest

The authors declare that the research was conducted in the absence of any commercial or financial relationships that could be construed as a potential conflict of interest.

## Publisher’s note

All claims expressed in this article are solely those of the authors and do not necessarily represent those of their affiliated organizations, or those of the publisher, the editors and the reviewers. Any product that may be evaluated in this article, or claim that may be made by its manufacturer, is not guaranteed or endorsed by the publisher.

## Abbreviations

AAV: adeno-associated virus
AMD: age-related macular degeneration
AON: antisense oligonucleotide
CGH: comparative genomic hybridization
CNV: copy number variations
CRISPR: clustered regularly interspaced short palindromic repeats
FISH: fluorescence *in situ* hybridization
IRD: inherited retinal diseases
LCA: Leber congenital amaurosis
LHON: Leber hereditary optic neuropathy
MLPA: multiplex ligation-dependent probe amplification
NGS: next generation sequencing
NP: nanoparticle
QF-PCR: quantitative fluorescent polymerase chain reaction
RGC: retinal ganglion cell
RP: retinitis pigmentosa
RPE: retinal pigment epithelium
USH: usher syndrome
VUS: variants of uncertain significance
WES: whole exome sequencing
WGS: whole genome sequencing
XLRS: X-linked retinoschisis.

## References

[1] Nash BM Wright DC Grigg JR Bennetts B Jamieson RV. Retinal dystrophies, genomic applications in diagnosis and prospects for therapy. *Transl Pediatr.* (2015) 4:139. 10.3978/J.ISSN.2224-4336.2015.04.03 26835369 PMC4729094

[2] Fenner BJ Tan TE Barathi AV Tun SBB Yeo SW Tsai ASH Gene-based therapeutics for inherited retinal diseases. *Front Genet.* (2022) 12:2743. 10.3389/FGENE.2021.794805/BIBTEX PMC8782148 35069693

[3] RetNet. *Summaries.* (2022). Available online at: https://sph.uth.edu/retnet/sum-dis.htm (accessed on March 16, 2022).

[4] Chen TC Huang DS Lin CW Yang CH Yang CM Wang VY Genetic characteristics and epidemiology of inherited retinal degeneration in Taiwan. *NPJ Genomic Med.* (2021) 6:16. 10.1038/s41525-021-00180-1 33608557 PMC7896090

[5] Vázquez-Domínguez I Garanto A Collin RWJ. Molecular therapies for inherited retinal diseases—current standing. *Opportunit Challeng Genes (Basel).* (2019) 10:654. 10.3390/GENES10090654 31466352 PMC6770110

[6] Moore NA Morral N Ciulla TA Bracha P. Gene therapy for inherited retinal and optic nerve degenerations. *Expert Opin Biol Ther.* (2018) 18:37–49. 10.1080/14712598.2018.1389886 29057663

[7] Tamhane M Cabrera-Ghayouri S Abelian G Viswanath V. Review of biomarkers in ocular matrices: challenges and opportunities. *Pharm Res.* (2019) 36:40. 10.1007/S11095-019-2569-8 30673862 PMC6344398

[8] Streilein JW. Ocular immune privilege: therapeutic opportunities from an experiment of nature. *Nat Rev Immunol.* (2003) 3:879–89. 10.1038/nri1224 14668804

[9] Zhou R Caspi RR. Ocular immune privilege. *F1000 Biol Rep.* (2010) 2:3. 10.3410/B2-3 20948803 PMC2948372

[10] Sugita S. Role of ocular pigment epithelial cells in immune privilege. *Arch Immunol Ther Exp.* (2009) 57:263–8. 10.1007/S00005-009-0030-0 19568919

[11] Kaur C Foulds WS Ling EA. Blood-retinal barrier in hypoxic ischaemic conditions: basic concepts, clinical features and management. *Prog Retin Eye Res.* (2008) 27:622–47. 10.1016/J.PRETEYERES.2008.09.003 18940262

[12] Simonelli F Maguire AM Testa F Pierce EA Mingozzi F Bennicelli JL Gene therapy for Leber’s congenital amaurosis is safe and effective through 1.5 years after vector administration. *Mol Ther.* (2010) 18:643. 10.1038/MT.2009.277 19953081 PMC2839440

[13] Martin KRG Klein RL Quigley HA. Gene delivery to the eye using adeno-associated viral vectors. *Methods.* (2002) 28:267–75. 10.1016/S1046-2023(02)00232-3 12413426

[14] Dhurandhar D Sahoo NK Mariappan I Narayanan R. Gene therapy in retinal diseases: a review. *Indian J Ophthalmol.* (2021) 69:2257–65. 10.4103/IJO.IJO_3117_20 34427196 PMC8544052

[15] Botto C Rucli M Tekinsoy MD Pulman J Sahel JA Dalkara D. Early and late stage gene therapy interventions for inherited retinal degenerations. *Prog Retin Eye Res.* (2022) 86:100975. 10.1016/J.PRETEYERES.2021.100975 34058340

[16] Gallego C Gonçalves MAFV Wijnholds J. Novel therapeutic approaches for the treatment of retinal degenerative diseases: focus on CRISPR/Cas-based gene editing. *Front Neurosci.* (2020) 14:838. 10.3389/FNINS.2020.00838/BIBTEX PMC7468381 32973430

[17] Ameri H. Prospect of retinal gene therapy following commercialization of voretigene neparvovec-rzyl for retinal dystrophy mediated by RPE65 mutation. *J Curr Ophthalmol.* (2018) 30:1. 10.1016/J.JOCO.2018.01.006 29564403 PMC5859497

[18] Amato A Arrigo A Aragona E Manitto MP Saladino A Bandello F Gene therapy in inherited retinal diseases: an update on current state of the art. *Front Med.* (2021) 8:750586. 10.3389/FMED.2021.750586 34722588 PMC8553993

[19] den Hollander AI. Omics in ophthalmology: advances in genomics and precision medicine for Leber congenital amaurosis and age-related macular degeneration. *Invest Ophthalmol Vis Sci.* (2016) 57:1378–87. 10.1167/IOVS.15-18167 27010695

[20] Singh M Tyagi SC. Genes and genetics in eye diseases: a genomic medicine approach for investigating hereditary and inflammatory ocular disorders. *Int J Ophthalmol.* (2018) 11:117. 10.18240/IJO.2018.01.20 29376001 PMC5767668

[21] Fritsche LG Fariss RN Stambolian D Abecasis GR Curcio CA Swaroop A. Age-related macular degeneration: genetics and biology coming together. *Annu Rev Genomics Hum Genet.* (2014) 15:151. 10.1146/ANNUREV-GENOM-090413-025610 24773320 PMC4217162

[22] Genetics and Health. *Genes, Behavior, and the Social Environment - NCBI Bookshelf.* (2022). Available online at: https://www.ncbi.nlm.nih.gov/books/NBK19932/ (accessed on May 28, 2022).

[23] Dockery A Whelan L Humphries P Jane Farrar G. Next-Generation sequencing applications for inherited retinal diseases. *Int J Mol Sci.* (2021) 22:5684. 10.3390/IJMS22115684 34073611 PMC8198572

[24] Diñeiro M Capín R Cifuentes G Fernández-Vega B Villota E Otero A Comprehensive genomic diagnosis of inherited retinal and optical nerve disorders reveals hidden syndromes and personalized therapeutic options. *Acta Ophthalmol.* (2020) 98:e1034. 10.1111/AOS.14479 32483926 PMC7754416

[25] Miraldi Utz V Coussa RG Antaki F Traboulsi EI. Gene therapy for RPE65-related retinal disease. *Ophthalmic Genet.* (2018) 39:671–7. 10.1080/13816810.2018.1533027 30335549

[26] Moosajee M Hingorani M Moore AT. *PAX6-Related Aniridia. GeneReviews§*. (2018). Available online at: https://www.ncbi.nlm.nih.gov/books/NBK1360/ (accessed on March 18, 2022).

[27] Heather JM Chain B. The sequence of sequencers: The history of sequencing DNA. *Genomics.* (2016) 107:1–8. 10.1016/J.YGENO.2015.11.003 26554401 PMC4727787

[28] Slatko BE Gardner AF Ausubel FM. Overview of next generation sequencing technologies. *Curr Protoc Mol Biol.* (2018) 122:e59. 10.1002/CPMB.59 29851291 PMC6020069

[29] Furey TS. ChIP-seq and beyond: new and improved methodologies to detect and characterize protein-DNA interactions. *Nat Rev Genet.* (2012) 13:840–52. 10.1038/NRG3306 23090257 PMC3591838

[30] Patel A Hayward JD Tailor V Nyanhete R Ahlfors H Gabriel C The Oculome panel test: next-generation sequencing to diagnose a diverse range of genetic developmental eye disorders. *Ophthalmology.* (2018) 126:888–907. 10.1016/j.ophtha.2018.12.050 30653986

[31] Jiman OA Taylor RL Lenassi E Smith JC Douzgou S Ellingford JM Diagnostic yield of panel-based genetic testing in syndromic inherited retinal disease. *Eur J Hum Genet.* (2020) 28:576–86. 10.1038/S41431-019-0548-5 31836858 PMC7171123

[32] Consugar MB Navarro-Gomez D Place EM Bujakowska KM Sousa ME Fonseca-Kelly ZD Panel-based genetic diagnostic testing for inherited eye diseases is highly accurate and reproducible, and more sensitive for variant detection, than exome sequencing. *Genet Med.* (2015) 17:253–61. 10.1038/GIM.2014.172 25412400 PMC4572572

[33] Sheck LHN Esposti SD Mahroo OA Arno G Pontikos N Wright G Panel-based genetic testing for inherited retinal disease screening 176 genes. *Mol Genet genomic Med.* (2021) 9:e1822. 10.1002/MGG3.1663 33749171 PMC8683638

[34] Numa S Oishi A Higasa K Oishi M Miyata M Hasegawa T EYS is a major gene involved in retinitis pigmentosa in Japan: genetic landscapes revealed by stepwise genetic screening. *Sci Rep.* (2020) 10:20770. 10.1038/S41598-020-77558-1 33247286 PMC7695703

[35] Van de Sompele S Smith C Karali M Corton M Van Schil K Peelman F Biallelic sequence and structural variants in RAX2 are a novel cause for autosomal recessive inherited retinal disease. *Genet Med.* (2019) 21:1319–29. 10.1038/S41436-018-0345-5 30377383 PMC6752271

[36] Avila-Fernandez A Corton M Nishiguchi KM Muñoz-Sanz N Benavides-Mori B Blanco-Kelly F Identification of an RP1 prevalent founder mutation and related phenotype in Spanish patients with early-onset autosomal recessive retinitis. *Ophthalmology.* (2012) 119:2616–21. 10.1016/J.OPHTHA.2012.06.033 22917891

[37] Perea-Romero I Gordo G Iancu IF Del Pozo-Valero M Almoguera B Blanco-Kelly F Genetic landscape of 6089 inherited retinal dystrophies affected cases in Spain and their therapeutic and extended epidemiological implications. *Sci Reports.* (2021) 11:1–13. 10.1038/s41598-021-81093-y 33452396 PMC7810997

[38] Tatour Y Tamaiev J Shamaly S Colombo R Bril E Rabinowitz T A novel intronic mutation of PDE6B is a major cause of autosomal recessive retinitis pigmentosa among Caucasus Jews. *Mol Vis.* (2019) 25:155. 30820151 PMC6386512

[39] Rabbani B Tekin M Mahdieh N. The promise of whole-exome sequencing in medical genetics. *J Hum Genet.* (2013) 59:5–15. 10.1038/jhg.2013.114 24196381

[40] Broadgate S Yu J Downes SM Halford S. Unravelling the genetics of inherited retinal dystrophies: past, present and future. *Prog Retin Eye Res.* (2017) 59:53–96. 10.1016/J.PRETEYERES.2017.03.003 28363849

[41] Méjécase C Mohand-Saïd S El Shamieh S Antonio A Condroyer C Blanchard S A novel nonsense variant in REEP6 is involved in a sporadic rod-cone dystrophy case. *Clin Genet.* (2018) 93:707–11. 10.1111/CGE.13171 29120066

[42] Arno G Agrawal SA Eblimit A Bellingham J Xu M Wang F Mutations in REEP6 cause autosomal-recessive retinitis pigmentosa. *Am J Hum Genet.* (2016) 99:1305–15. 10.1016/J.AJHG.2016.10.008 27889058 PMC5142109

[43] Hull S Owen N Islam F Tracey-White D Plagnol V Holder GE Nonsyndromic retinal dystrophy due to bi-allelic mutations in the ciliary transport gene IFT140. *Invest Ophthalmol Vis Sci.* (2016) 57:1053–62. 10.1167/iovs.15-17976 26968735

[44] Adams DR Eng CM. Next-generation sequencing to diagnose suspected genetic disorders. *N Engl J Med.* (2018) 379:1353–62. 10.1056/nejmra1711801 30281996

[45] Lelieveld SH Spielmann M Mundlos S Veltman JA Gilissen C. Comparison of exome and genome sequencing technologies for the complete capture of protein-coding regions. *Hum Mutat.* (2015) 36:815–22. 10.1002/HUMU.22813 25973577 PMC4755152

[46] Meienberg J Bruggmann R Oexle K Matyas G. Clinical sequencing: is WGS the better WES? *Hum Genet.* (2016) 135:359–62. 10.1007/S00439-015-1631-9 26742503 PMC4757617

[47] Bentley DR Balasubramanian S Swerdlow HP Smith GP Milton J Brown CG Accurate whole human genome sequencing using reversible terminator chemistry. *Nature.* (2008) 456:53–9. 10.1038/NATURE07517 18987734 PMC2581791

[48] Kozarewa I Ning Z Quail MA Sanders MJ Berriman M Turner DJ. Amplification-free Illumina sequencing-library preparation facilitates improved mapping and assembly of (G+C)-biased genomes. *Nat Methods.* (2009) 6:291–5. 10.1038/NMETH.1311 19287394 PMC2664327

[49] Lionel AC Costain G Monfared N Walker S Reuter MS Hosseini SM Improved diagnostic yield compared with targeted gene sequencing panels suggests a role for whole-genome sequencing as a first-tier genetic test. *Genet Med.* (2018) 20:435–43. 10.1038/GIM.2017.119 28771251 PMC5895460

[50] Van Cauwenbergh C Van Schil K Cannoodt R Bauwens M Van Laethem T De Jaegere S arrEYE: a customized platform for high-resolution copy number analysis of coding and noncoding regions of known and candidate retinal dystrophy genes and retinal noncoding RNAs. *Genet Med.* (2017) 19:457–66. 10.1038/GIM.2016.119 27608171 PMC5392597

[51] Carss K Arno G Erwood M Stephens J Sanchis-Juan A Hull S Comprehensive rare variant analysis via whole-genome sequencing to determine the molecular pathology of inherited retinal disease. *Am J Hum Genet.* (2017) 100:75–90. 10.1016/J.AJHG.2016.12.003 28041643 PMC5223092

[52] Speicher MR Carter NP. The new cytogenetics: blurring the boundaries with molecular biology. *Nat Rev Genet.* (2005) 6:782–92. 10.1038/NRG1692 16145555

[53] Tanimoto K Sekiguchi N Yokota Y Kaneko A Watanabe T Maeshima AM Fluorescence in situ hybridization (FISH) analysis of primary ocular adnexal MALT lymphoma. *BMC Cancer.* (2006) 6:249. 10.1186/1471-2407-6-249 17052360 PMC1630703

[54] Singh AD Aronow ME Sun Y Bebek G Saunthararajah Y Schoenfield LR Chromosome 3 status in uveal melanoma: a comparison of fluorescence in situ hybridization and single-nucleotide polymorphism array. *Invest Ophthalmol Vis Sci.* (2012) 53:3331–9. 10.1167/IOVS.11-9027 22511634 PMC4625803

[55] Smeets DFCM. Historical prospective of human cytogenetics: from microscope to microarray. *Clin Biochem.* (2004) 37:439–46. 10.1016/J.CLINBIOCHEM.2004.03.006 15183291

[56] Dugoff L Norton ME Kuller JA. The use of chromosomal microarray for prenatal diagnosis. *Am J Obstet Gynecol.* (2016) 215:B2–9. 10.1016/J.AJOG.2016.07.016 27427470

[57] Stirn Kranjc B. Ocular abnormalities and systemic disease in Down syndrome. *Strabismus.* (2012) 20:74–7. 10.3109/09273972.2012.680234 22612356

[58] Margaret Woodhouse J Pakeman VH Cregg M Saunders KJ Parker M Fraser WI Refractive errors in young children with Down syndrome. *Optom Vis Sci.* (1997) 74:844–51. 10.1097/00006324-199710000-00023 9383798

[59] Wagner RS Caputo AR Reynolds RD. Nystagmus in Down’s syndrome. *Ophthalmology.* (1990) 97:1439–44. 10.1016/S0161-6420(90)32399-0 2147744

[60] Shaw-Smith C Redon R Rickman L Rio M Willatt L Fiegler H Microarray based comparative genomic hybridisation (array-CGH) detects submicroscopic chromosomal deletions and duplications in patients with learning disability/mental retardation and dysmorphic features. *J Med Genet.* (2004) 41:241–8. 10.1136/JMG.2003.017731 15060094 PMC1735726

[61] Richardson R Hingorani M Van Heyningen V Gregory-Evans C Moosajee M. Clinical utility gene card for: Aniridia. *Eur J Hum Genet.* (2016) 24:24. 10.1038/EJHG.2016.73 27381094 PMC5110069

[62] Cirigliano V Ejarque M Cañadas MP Lloveras E Plaja A Del Mar Perez M Clinical application of multiplex quantitative fluorescent polymerase chain reaction (QF-PCR) for the rapid prenatal detection of common chromosome aneuploidies. *Mol Hum Reprod.* (2001) 7:1001–6. 10.1093/MOLEHR/7.10.1001 11574670

[63] Lee C Iafrate AJ Brothman AR. Copy number variations and clinical cytogenetic diagnosis of constitutional disorders. *Nat Genet.* (2007) 39:S48–54. 10.1038/NG2092 17597782

[64] Schouten JP McElgunn CJ Waaijer R Zwijnenburg D Diepvens F Pals G. Relative quantification of 40 nucleic acid sequences by multiplex ligation-dependent probe amplification. *Nucleic Acids Res.* (2002) 30:e57. 10.1093/NAR/GNF056 12060695 PMC117299

[65] Redeker EJW de Visser ASH Bergen AAB Mannens MMAM. Multiplex ligation-dependent probe amplification (MLPA) enhances the molecular diagnosis of aniridia and related disorders. *Mol Vis.* (2008) 14:836. 18483559 PMC2375324

[66] Gupta H Malaichamy S Mallipatna A Murugan S Jeyabalan N Suresh Babu V Retinoblastoma genetics screening and clinical management. *BMC Med Genom.* (2021) 14:188. 10.1186/S12920-021-01034-6 34294096 PMC8296631

[67] Chiang JPW Trzupek K. The current status of molecular diagnosis of inherited retinal dystrophies. *Curr Opin Ophthalmol.* (2015) 26:346–51. 10.1097/ICU.0000000000000185 26214332

[68] Wang F Wang H Tuan HF Nguyen DH Sun V Keser V Next generation sequencing-based molecular diagnosis of retinitis pigmentosa: identification of a novel genotype-phenotype correlation and clinical refinements. *Hum Genet.* (2014) 133:331. 10.1007/S00439-013-1381-5 24154662 PMC3945441

[69] Stone EM Aldave AJ Drack AV MacCumber MW Sheffield VC Traboulsi E Recommendations for genetic testing of inherited eye diseases: report of the American academy of ophthalmology task force on genetic testing. *Ophthalmology.* (2012) 119:2408–10. 10.1016/J.OPHTHA.2012.05.047 22944025

[70] Raspa M Moultrie R Toth D Haque SN. Barriers and facilitators to genetic service delivery models: scoping review. *Interact J Med Res.* (2021) 10:e23523. 10.2196/23523 33629958 PMC7952239

[71] NSGC. (2022). Available online at: https://www.nsgc.org/ (accessed on March 17, 2022).

[72] Rathi S Tsui E Mehta N Zahid S Schuman JS. The current state of teleophthalmology in the United States. *Ophthalmology.* (2017) 124:1729. 10.1016/J.OPHTHA.2017.05.026 28647202 PMC6020848

[73] Black GC Sergouniotis P Sodi A Leroy BP Van Cauwenbergh C Liskova P The need for widely available genomic testing in rare eye diseases: an ERN-EYE position statement. *Orphanet J Rare Dis.* (2021) 16:142. 10.1186/S13023-021-01756-X 33743793 PMC7980559

[74] Testing G. *Help Me Understand Genetics.* (2022). Available online at: https://medlineplus.gov/genetics/ (accessed on March 17, 2022).

[75] Lam BL Leroy BP Black G Ong T Yoon D Trzupek K. Genetic testing and diagnosis of inherited retinal diseases. *Orphanet J Rare Dis.* (2021) 16:514. 10.1186/S13023-021-02145-0/TABLES/2 PMC8670140 34906171

[76] Lee K Garg S. Navigating the current landscape of clinical genetic testing for inherited retinal dystrophies. *Genet Med.* (2015) 17:245–52. 10.1038/GIM.2015.15 25790163

[77] Foundation Fighting Blindness. *Genetic Testing For Retinal Degenerative Diseases: Information and Resources for Affected Individuals, Families and Health Care Providers.* (2022). Available online at: https://www.fightingblindness.org/genetic-testing-for-retinal-degenerative-diseases-information-and-resources-for-affected-individuals-families-and-health-care-providers (accessed on March 17, 2022).

[78] Blain D Brooks BP. Molecular diagnosis and genetic counseling in ophthalmology. *Arch Ophthalmol.* (2007) 125:196–203. 10.1001/ARCHOPHT.125.2.196 17296895

[79] Yohe S Sivasankar M Ghosh A Ghosh A Holle J Murugan S Prevalence of mutations in inherited retinal diseases: a comparison between the United States and India. *Mol Genet genomic Med.* (2020) 8:e1081. 10.1002/MGG3.1081 31816670 PMC7005662

[80] Wall JD Stawiski EW Ratan A Kim HL Kim C Gupta R The genomeAsia 100K project enables genetic discoveries across Asia. *Nature.* (2019) 576:106–11. 10.1038/S41586-019-1793-Z 31802016 PMC7054211

[81] Richards S Aziz N Bale S Bick D Das S Gastier-Foster J Standards and guidelines for the interpretation of sequence variants: a joint consensus recommendation of the American college of medical genetics and genomics and the association for molecular pathology. *Genet Med.* (2015) 17:405–24. 10.1038/GIM.2015.30 25741868 PMC4544753

[82] Zhang JX Wang NL Lu QJ. Development of gene and stem cell therapy for ocular neurodegeneration. *Int J Ophthalmol.* (2015) 8:622. 10.3980/J.ISSN.2222-3959.2015.03.33 26086019 PMC4458674

[83] Gordon K Del Medico A Sander I Kumar A Hamad B. Gene therapies in ophthalmic disease. *Nat Rev Drug Discov.* (2019) 18:415–6. 10.1038/D41573-018-00016-1 31160760

[84] Winkler PA Occelli LM Petersen-Jones SM. Large animal models of inherited retinal degenerations: a review. *Cells.* (2020) 9:882. 10.3390/CELLS9040882 32260251 PMC7226744

[85] Lee JH Wang JH Chen J Li F Edwards TL Hewitt AW Gene therapy for visual loss: opportunities and concerns. *Prog Retin Eye Res.* (2019) 68:31–53. 10.1016/J.PRETEYERES.2018.08.003 30170104

[86] Hu ML Edwards TL O’Hare F Hickey DG Wang JH Liu Z Gene therapy for inherited retinal diseases: progress and possibilities. *Clin Exp Optom.* (2021) 104:444–54. 10.1080/08164622.2021.1880863 33689657

[87] Kohl S Jägle H Wissinger B Zobor D. *Achromatopsia. GeneReviews§*. (2018). Available online at: https://www.ncbi.nlm.nih.gov/books/NBK1418/ (accessed on March 17, 2022).

[88] Komáromy AM Alexander JJ Rowlan JS Garcia MM Chiodo VA Kaya A Gene therapy rescues cone function in congenital achromatopsia. *Hum Mol Genet.* (2010) 19:2581. 10.1093/HMG/DDQ136 20378608 PMC2883338

[89] Ofri R Averbukh E Ezra-Elia R Ross M Honig H Obolensky A Six years and counting: restoration of photopic retinal function and visual behavior following gene augmentation therapy in a sheep model of CNGA3 achromatopsia. *Hum Gene Ther.* (2018) 29:1376–86. 10.1089/HUM.2018.076 29926749 PMC12225372

[90] Michalakis S Mühlfriedel R Tanimoto N Krishnamoorthy V Koch S Fischer MD Restoration of cone vision in the CNGA3-/- mouse model of congenital complete lack of cone photoreceptor function. *Mol Ther.* (2010) 18:2057. 10.1038/MT.2010.149 20628362 PMC2997579

[91] Bush RA Zeng Y Colosi P Kjellstrom S Hiriyanna S Vijayasarathy C Preclinical dose-escalation study of intravitreal AAV-RS1 gene therapy in a mouse model of X-linked retinoschisis: Dose-dependent expression and improved retinal structure and function. *Hum Gene Ther.* (2016) 27:376–89. 10.1089/HUM.2015.142 27036983 PMC4840830

[92] Koponen S Kokki E Kinnunen K Ylä-Herttuala S. Viral-vector-delivered anti-angiogenic therapies to the eye. *Pharmaceutics.* (2021) 13:219. 10.3390/PHARMACEUTICS13020219 33562561 PMC7915489

[93] Bouquet C Vignal Clermont C Galy A Fitoussi S Blouin L Munk MR Immune response and intraocular inflammation in patients with leber hereditary optic neuropathy treated with intravitreal injection of recombinant adeno-associated virus 2 carrying the ND4 gene: a secondary analysis of a phase 1/2 clinical trial. *JAMA Ophthalmol.* (2019) 137:399. 10.1001/JAMAOPHTHALMOL.2018.6902 30730541 PMC6459107

[94] Burnight ER Giacalone JC Cooke JA Thompson JR Bohrer LR Chirco KR CRISPR-Cas9 genome engineering: Treating inherited retinal degeneration. *Prog Retin Eye Res.* (2018) 65:28–49. 10.1016/J.PRETEYERES.2018.03.003 29578069 PMC8210531

[95] ClinicalTrials.gov. *Single Ascending Dose Study in Participants With LCA10 - Full Text View.* (2022). Available online at: https://clinicaltrials.gov/ct2/show/NCT03872479 (accessed on March 18, 2022).

[96] Ledford H. CRISPR treatment inserted directly into the body for first time. *Nature.* (2020) 579:185. 10.1038/D41586-020-00655-8 32157225

[97] Maeder ML Stefanidakis M Wilson CJ Baral R Barrera LA Bounoutas GS Development of a gene-editing approach to restore vision loss in Leber congenital amaurosis type 10. *Nat Med.* (2019) 25:229–33. 10.1038/S41591-018-0327-9 30664785

[98] Editas Medicine. *Allergan and Editas Medicine Announce Dosing of First Patient in Landmark Phase 1/2 Clinical Trial of CRISPR Medicine AGN-151587 (EDIT-101) for the Treatment of LCA10.* (2022). Available online at: https://ir.editasmedicine.com/news-releases/news-release-details/allergan-and-editas-medicine-announce-dosing-first-patient (accessed on March 18, 2022).

[99] Bennett Frank C Krainer AR Cleveland DW. Antisense oligonucleotide therapies for neurodegenerative diseases. *Annu Rev Neurosci.* (2019) 42:385–406. 10.1146/ANNUREV-NEURO-070918-050501 31283897 PMC7427431

[100] Van Den Hurk JAJM Van De Pol DJR Wissinger B Van Driel MA Hoefsloot LH De Wijs IJ Novel types of mutation in the choroideremia (CHM) gene: a full-length L1 insertion and an intronic mutation activating a cryptic exon. *Hum Genet.* (2003) 113:268–75. 10.1007/S00439-003-0970-0 12827496

[101] Garanto A van der Velde-Visser SD Cremers FPM Collin RWJ. Antisense oligonucleotide-based splice correction of a deep-intronic mutation in CHM underlying choroideremia. *Adv Exp Med Biol.* (2018) 1074:83–9. 10.1007/978-3-319-75402-4_11 29721931

[102] Aukrust I Jansson RW Bredrup C Rusaas HE Berland S Jørgensen A The intronic ABCA4 c.5461-10T>C variant, frequently seen in patients with Stargardt disease, causes splice defects and reduced ABCA4 protein level. *Acta Ophthalmol.* (2017) 95:240–6. 10.1111/AOS.13273 27775217

[103] Sangermano R Garanto A Khan M Runhart EH Bauwens M Bax NM Deep-intronic ABCA4 variants explain missing heritability in Stargardt disease and allow correction of splice defects by antisense oligonucleotides. *Genet Med.* (2019) 21:1751–60. 10.1038/S41436-018-0414-9 30643219 PMC6752325

[104] Cideciyan AV Jacobson SG Drack AV Ho AC Charng J Garafalo AV Effect of an intravitreal antisense oligonucleotide on vision in Leber congenital amaurosis due to a photoreceptor cilium defect. *Nat Med.* (2019) 25:225–8. 10.1038/S41591-018-0295-0 30559420

[105] Lenassi E Saihan Z Bitner-Glindzicz M Webster AR. The effect of the common c.2299delG mutation in USH2A on RNA splicing. *Exp Eye Res.* (2014) 122:9–12. 10.1016/J.EXER.2014.02.018 24607488

[106] Solano ECR Kornbrust DJ Beaudry A Foy JWD Schneider DJ Thompson JD. Toxicological and pharmacokinetic properties of QPI-1007, a chemically modified synthetic siRNA targeting caspase 2 mRNA, following intravitreal injection. *Nucleic Acid Ther.* (2014) 24:258–66. 10.1089/NAT.2014.0489 25054518

[107] Cideciyan AV Jacobson SG Beltran WA Sumaroka A Swider M Iwabe S Human retinal gene therapy for Leber congenital amaurosis shows advancing retinal degeneration despite enduring visual improvement. *Proc Natl Acad Sci USA.* (2013) 110:E517–25. 10.1073/PNAS.1218933110 23341635 PMC3568385

[108] Bainbridge JWB Smith AJ Barker SS Robbie S Henderson R Balaggan K Effect of gene therapy on visual function in Leber’s congenital amaurosis. *N Engl J Med.* (2008) 358:2231–9. 10.1056/NEJMOA0802268 18441371

[109] Jacobson SG Cideciyan AV Roman AJ Sumaroka A Schwartz SB Heon E Improvement and decline in vision with gene therapy in childhood blindness. *N Engl J Med.* (2015) 372:1920–6. 10.1056/NEJMOA1412965/SUPPL_FILE/NEJMOA1412965_DISCLOSURES.PDF 25936984 PMC4450362

[110] Lo ACY Woo TTY Wong RLM Wong D. Apoptosis and other cell death mechanisms after retinal detachment: implications for photoreceptor rescue. *Ophthalmologica.* (2011) 226 (Suppl. 1):10–7. 10.1159/000328206 21778775

[111] Wu L Ueda K Nagasaki T Sparrow JR. Light damage in Abca4 and Rpe65rd12 Mice. *Invest Ophthalmol Vis Sci.* (2014) 55:1910–8. 10.1167/IOVS.14-13867 24576873 PMC3973190

[112] Sawada O Perusek L Kohno H Howell SJ Maeda A Matsuyama S All-trans-retinal induces Bax activation via DNA damage to mediate retinal cell apoptosis. *Exp Eye Res.* (2014) 123:27–36. 10.1016/J.EXER.2014.04.003 24726920 PMC4083191

[113] Hamann S Schorderet DF Cottet S. Bax-induced apoptosis in Leber’s congenital amaurosis: a dual role in rod and cone degeneration. *PLoS One.* (2009) 4:e6616. 10.1371/JOURNAL.PONE.0006616 19672311 PMC2720534

[114] Garafalo AV Cideciyan AV Héon E Sheplock R Pearson A WeiYang Yu C Progress in treating inherited retinal diseases: early subretinal gene therapy clinical trials and candidates for future initiatives. *Prog Retin Eye Res.* (2020) 77:100827. 10.1016/J.PRETEYERES.2019.100827 31899291 PMC8714059

[115] Remé CE Grimm C Hafezi F Wenzel A Williams TP. Apoptosis in the retina: the silent death of vision. *News Physiol Sci.* (2000) 15:120–5. 10.1152/PHYSIOLOGYONLINE.2000.15.3.120 11390893

[116] Murakami Y Ikeda Y Nakatake S Miller JW Vavvas DG Sonoda KH Necrotic cone photoreceptor cell death in retinitis pigmentosa. *Cell Death Dis.* (2015) 6:e2038. 10.1038/CDDIS.2015.385 26720347 PMC4720913

[117] McIlwain DR Berger T Mak TW. Caspase functions in cell death and disease. *Cold Spring Harb Perspect Biol.* (2013) 5:a008656. 10.1101/CSHPERSPECT.A008656 23545416 PMC3683896

[118] Silke J Vucic D. IAP family of cell death and signaling regulators. *Methods Enzymol.* (2014) 545:35–65. 10.1016/B978-0-12-801430-1.00002-0 25065885

[119] Hua P Liu LB Liu JL Wang M Jiang HQ Zeng K Inhibition of apoptosis by knockdown of caspase-3 with siRNA in rat bone marrow mesenchymal stem cells. *Exp Biol Med (Maywood).* (2013) 238:991–8. 10.1177/1535370213497320 23900153

[120] Kosmaoglou M Schwarz N Bett JS Cheetham ME. Molecular chaperones and photoreceptor function. *Prog Retin Eye Res.* (2008) 27:434. 10.1016/J.PRETEYERES.2008.03.001 18490186 PMC2568879

[121] Kimura A Namekata K Guo X Harada C Harada T. Neuroprotection, growth factors and BDNF-TrkB signalling in retinal degeneration. *Int J Mol Sci.* (2016) 17:1584. 10.3390/IJMS17091584 27657046 PMC5037849

[122] Lenzi L Coassin M Lambiase A Bonini S Amendola T Aloe L. Effect of exogenous administration of nerve growth factorin the retina of rats with inherited retinitis pigmentosa. *Vision Res.* (2005) 45:1491–500. 10.1016/J.VISRES.2004.12.020 15781068

[123] Wu SM Hochedlinger K. Harnessing the potential of induced pluripotent stem cells for regenerative medicine. *Nat Cell Biol.* (2011) 13:497. 10.1038/NCB0511-497 21540845 PMC3617981

[124] Lamas NJ Kerner BJ Roybon L Kim YA Diaz AG Wichterle H Neurotrophic requirements of human motor neurons defined using amplified and purified stem cell-derived cultures. *PLoS One.* (2014) 9:e110324. 10.1371/JOURNAL.PONE.0110324 25337699 PMC4206291

[125] Roybon L Lamas NJ Garcia-Diaz A Yang EJ Sattler R Jackson-Lewis V Human stem cell-derived spinal cord astrocytes with defined mature or reactive phenotypes. *Cell Rep.* (2013) 4:1035–48. 10.1016/J.CELREP.2013.06.021 23994478 PMC4229657

[126] Wichterle H Lieberam I Porter JA Jessell TM. Directed differentiation of embryonic stem cells into motor neurons. *Cell.* (2002) 110:385–97. 10.1016/S0092-8674(02)00835-8 12176325

[127] Anderson DR. Collaborative normal tension glaucoma study. *Curr Opin Ophthalmol.* (2003) 14:86–90. 10.1097/00055735-200304000-00006 12698048

[128] Quigley H Broman AT. The number of people with glaucoma worldwide in 2010 and 2020. *Br J Ophthalmol.* (2006) 90:262–7. 10.1136/BJO.2005.081224 16488940 PMC1856963

[129] Johnson TV Bull ND Martin KR. Neurotrophic factor delivery as a protective treatment for glaucoma. *Exp Eye Res.* (2011) 93:196–203. 10.1016/J.EXER.2010.05.016 20685205

[130] Unsicker K. Neurotrophic molecules in the treatment of neurodegenerative disease with focus on the retina: status and perspectives. *Cell Tissue Res.* (2013) 353:205–18. 10.1007/S00441-013-1585-Y 23463189

[131] Paulus YM Campbell JP. Neuroprotection and retinal diseases. *Dev Ophthalmol.* (2016) 55:322–9. 10.1159/000434703 26501142

[132] Pardue MT Allen RS. Neuroprotective strategies for retinal disease. *Prog Retin Eye Res.* (2018) 65:50–76. 10.1016/J.PRETEYERES.2018.02.002 29481975 PMC6081194

[133] Osborne A Khatib TZ Songra L Barber AC Hall K Kong GYX Neuroprotection of retinal ganglion cells by a novel gene therapy construct that achieves sustained enhancement of brain-derived neurotrophic factor/tropomyosin-related kinase receptor-B signaling. *Cell Death Dis.* (2018) 9:1007. 10.1038/S41419-018-1041-8 30258047 PMC6158290

[134] Duebel J Marazova K Sahel JA. Optogenetics. *Curr Opin Ophthalmol.* (2015) 26:226–32. 10.1097/ICU.0000000000000140 25759964 PMC5395664

[135] McClements ME Staurenghi F MacLaren RE Cehajic-Kapetanovic J. Optogenetic gene therapy for the degenerate retina: recent advances. *Front Neurosci.* (2020) 14:1187. 10.3389/FNINS.2020.570909/BIBTEX PMC7686539 33262683

[136] Sahel JA Boulanger-Scemama E Pagot C Arleo A Galluppi F Martel JN Partial recovery of visual function in a blind patient after optogenetic therapy. *Nat Med.* (2021) 27:1223–9. 10.1038/s41591-021-01351-4 34031601

[137] Simon C-J Chaol A Grimaud A Eickelbeck D Rucli M. Reactivating the phototransduction cascade by universally applicable gene therapy preserves retinal function in Rod-Cone dystrophy. *Res Sq.* (2021). 10.21203/rs.3.rs-1189099/v1

[138] Simunovic MP Shen W Lin JY Protti DA Lisowski L Gillies MC. Optogenetic approaches to vision restoration. *Exp Eye Res.* (2019) 178:15–26. 10.1016/J.EXER.2018.09.003 30218651

[139] Ramamoorth M Narvekar A. Non viral vectors in gene therapy- an overview. *J Clin Diagn Res.* (2015) 9:GE01. 10.7860/JCDR/2015/10443.5394 25738007 PMC4347098

[140] Ziccardi L Cordeddu V Gaddini L Matteucci A Parravano M Malchiodi-Albedi F Gene therapy in retinal dystrophies. *Int J Mol Sci.* (2019) 20:5722. 10.3390/IJMS20225722 31739639 PMC6888000

[141] Apte RS. Gene therapy for retinal degeneration. *Cell* (2018) 173:5. 10.1016/J.CELL.2018.03.021 29570997

[142] Surace EM Auricchio A. Versatility of AAV vectors for retinal gene transfer. *Vision Res.* (2008) 48:353–9. 10.1016/J.VISRES.2007.07.027 17923143

[143] Srivastava A. In vivo tissue-tropism of adeno-associated viral vectors. *Curr Opin Virol.* (2016) 21:75–80. 10.1016/J.COVIRO.2016.08.003 27596608 PMC5138125

[144] Patrício MI Barnard AR Xue K MacLaren RE. Choroideremia: molecular mechanisms and development of AAV gene therapy. *Expert Opin Biol Ther.* (2018) 18:807–20. 10.1080/14712598.2018.1484448 29932012

[145] Auricchio A Kobinger G Anand V Hildinger M O’Connor E Maguire AM Exchange of surface proteins impacts on viral vector cellular specificity and transduction characteristics: the retina as a model. *Hum Mol Genet.* (2001) 10:3075–81. 10.1093/HMG/10.26.3075 11751689

[146] Rabinowitz JE Rolling F Li C Conrath H Xiao W Xiao X Cross-packaging of a single adeno-associated virus (AAV) type 2 vector genome into multiple AAV serotypes enables transduction with broad specificity. *J Virol.* (2002) 76:791–801. 10.1128/JVI.76.2.791-801.2002 11752169 PMC136844

[147] Yang GS Schmidt M Yan Z Lindbloom JD Harding TC Donahue BA Virus-mediated transduction of murine retina with adeno-associated virus: effects of viral capsid and genome size. *J Virol.* (2002) 76:7651–60. 10.1128/JVI.76.15.7651-7660.2002 12097579 PMC136354

[148] Lebherz C Maguire A Tang W Bennett J Wilson JM. Novel AAV serotypes for improved ocular gene transfer. *J Gene Med.* (2008) 10:375–82. 10.1002/JGM.1126 18278824 PMC2842078

[149] Petrs-Silva H Dinculescu A Li Q Deng WT Pang JJ Min SH Novel properties of tyrosine-mutant AAV2 vectors in the mouse retina. *Mol Ther.* (2011) 19:293–301. 10.1038/MT.2010.234 21045809 PMC3034844

[150] Petrs-Silva H Dinculescu A Li Q Min SH Chiodo V Pang JJ High-efficiency transduction of the mouse retina by tyrosine-mutant AAV serotype vectors. *Mol Ther.* (2009) 17:463–71. 10.1038/MT.2008.269 19066593 PMC2835095

[151] Mowat FM Gornik KR Dinculescu A Boye SL Hauswirth WW Petersen-Jones SM Tyrosine capsid-mutant AAV vectors for gene delivery to the canine retina from a subretinal or intravitreal approach. *Gene Ther.* (2014) 21:96. 10.1038/GT.2013.64 24225638 PMC3880610

[152] Flannery JG Visel M. Adeno-associated viral vectors for gene therapy of inherited retinal degenerations. *Methods Mol Biol.* (2013) 935:351–69. 10.1007/978-1-62703-080-9_25 23150381

[153] Trapani I Colella P Sommella A Iodice C Cesi G de Simone S Effective delivery of large genes to the retina by dual AAV vectors. *EMBO Mol Med.* (2014) 6:194–211. 10.1002/EMMM.201302948 24150896 PMC3927955

[154] Colella P Trapani I Cesi G Sommella A Manfredi A Puppo A Efficient gene delivery to the cone-enriched pig retina by dual AAV vectors. *Gene Ther.* (2014) 21:450–6. 10.1038/GT.2014.8 24572793

[155] Ghosh A Yue Y Long C Bostick B Duan D. Efficient whole-body transduction with trans-splicing adeno-associated viral vectors. *Mol Ther.* (2007) 15:750–5. 10.1038/SJ.MT.6300081 17264855 PMC2581720

[156] Ghosh A Yue Y Lai Y Duan D. A hybrid vector system expands adeno-associated viral vector packaging capacity in a transgene-independent manner. *Mol Ther.* (2008) 16:124–30. 10.1038/SJ.MT.6300322 17984978

[157] Yue Y Ghosh A Long C Bostick B Smith BF Kornegay JN A single intravenous injection of adeno-associated virus serotype-9 leads to whole body skeletal muscle transduction in dogs. *Mol Ther.* (2008) 16:1944–52. 10.1038/MT.2008.207 18827804 PMC2703820

[158] Lopes VS Boye SE Louie CM Boye S Dyka F Chiodo V Retinal gene therapy with a large MYO7A cDNA using adeno-associated virus. *Gene Ther.* (2013) 20:824–33. 10.1038/GT.2013.3 23344065 PMC3640772

[159] Trapani I. Dual AAV Vectors for Stargardt Disease. *Methods Mol Biol.* (2018) 1715:153–75. 10.1007/978-1-4939-7522-8_11 29188512

[160] Maddalena A Tornabene P Tiberi P Minopoli R Manfredi A Mutarelli M Triple vectors expand AAV transfer capacity in the retina. *Mol Ther.* (2018) 26:524. 10.1016/J.YMTHE.2017.11.019 29292161 PMC5835116

[161] Trapani I Puppo A Auricchio A. Vector platforms for gene therapy of inherited retinopathies. *Prog Retin Eye Res.* (2014) 43:108–28. 10.1016/J.PRETEYERES.2014.08.001 25124745 PMC4241499

[162] Bordet T Behar-Cohen F. Ocular gene therapies in clinical practice: viral vectors and nonviral alternatives. *Drug Discov Today.* (2019) 24:1685–93. 10.1016/J.DRUDIS.2019.05.038 31173914

[163] Tsai CH Wang PY Lin IC Huang H Liu GS Tseng CL. Ocular drug delivery: role of degradable polymeric nanocarriers for ophthalmic application. *Int J Mol Sci.* (2018) 19:2830. 10.3390/IJMS19092830 30235809 PMC6164366

[164] Masse F Ouellette M Lamoureux G Boisselier E. Gold nanoparticles in ophthalmology. *Med Res Rev.* (2019) 39:302–27. 10.1002/MED.21509 29766541

[165] Wang Y Rajala A Rajala RVS. Lipid nanoparticles for ocular gene delivery. *J Funct Biomater.* (2015) 6:379. 10.3390/JFB6020379 26062170 PMC4493518

[166] Battaglia L Serpe L Foglietta F Muntoni E Gallarate M Del Pozo Rodriguez A Application of lipid nanoparticles to ocular drug delivery. *Expert Opin Drug Deliv.* (2016) 13:1743–57. 10.1080/17425247.2016.1201059 27291069

[167] Oliveira AV Rosa da Costa AM Silva GA. Non-viral strategies for ocular gene delivery. *Mater Sci Eng C Mater Biol Appl.* (2017) 77:1275–89. 10.1016/J.MSEC.2017.04.068 28532005

[168] Zulliger R Conley SM Naash MI. Non-viral therapeutic approaches to ocular diseases: an overview and future directions. *J Contr Rel.* (2015) 219:471. 10.1016/J.JCONREL.2015.10.007 26439665 PMC4699668

[169] Adijanto J Naash MI. Nanoparticle-based technologies for retinal gene therapy. *Eur J Pharm Biopharm.* (2015) 95:353. 10.1016/J.EJPB.2014.12.028 25592325 PMC4499495

[170] Chen X Kube DM Cooper MJ Davis PB. Cell surface nucleolin serves as receptor for DNA nanoparticles composed of pegylated polylysine and DNA. *Mol Ther.* (2008) 16:333–42. 10.1038/SJ.MT.6300365/ATTACHMENT/AF9198EF-D1E6-4BC6-89E9-D72BE35CC5F9/MMC4.DOC 18059369

[171] Koirala A Makkia RS Conley SM Cooper MJ Naash MIS. /MAR-containing DNA nanoparticles promote persistent RPE gene expression and improvement in RPE65-associated LCA. *Hum Mol Genet.* (2013) 22:1632–42. 10.1093/HMG/DDT013 23335596 PMC3605833

[172] Han Z Koirala A Makkia R Cooper MJ Naash MI. Direct gene transfer with compacted DNA nanoparticles in retinal pigment epithelial cells: expression, repeat delivery and lack of toxicity. *Nanomedicine (Lond).* (2012) 7:521–39. 10.2217/NNM.11.158 22356602 PMC3404893

[173] Akelley RA Conley SM Makkia R Watson JN Han Z Cooper MJ DNA nanoparticles are safe and nontoxic in non-human primate eyes. *Int J Nanomed.* (2018) 13:1361. 10.2147/IJN.S157000 29563793 PMC5849385

[174] Andrieu-Soler C Halhal M Boatright JH Padove SA Nickerson JM Stodulkova E Single-stranded oligonucleotide-mediated in vivo gene repair in the rd1 retina. *Mol Vis.* (2007) 13:692. 17563719 PMC2765472

[175] Souied EH Reid SNM Piri NI Lerner LE Nusinowitz S Farber DB. Non-invasive gene transfer by iontophoresis for therapy of an inherited retinal degeneration. *Exp Eye Res.* (2008) 87:168–75. 10.1016/J.EXER.2008.04.009 18653181 PMC2713253

[176] Sharma A Tandon A Tovey JCK Gupta R Robertson JD Fortune JA Polyethylenimine-conjugated gold nanoparticles: Gene transfer potential and low toxicity in the cornea. *Nanomedicine.* (2011) 7:505–13. 10.1016/J.NANO.2011.01.006 21272669 PMC3094737

[177] Cervia LD Yuan F. Current progress in electrotransfection as a nonviral method for gene delivery. *Mol Pharm.* (2018) 15:3617–24. 10.1021/ACS.MOLPHARMACEUT.8B00207 29889538 PMC6123289

[178] Czugala M Mykhaylyk O Böhler P Onderka J Stork B Wesselborg S Efficient and safe gene delivery to human corneal endothelium using magnetic nanoparticles. *Nanomedicine (Lond).* (2016) 11:1787–800. 10.2217/NNM-2016-0144 27388974

[179] Batabyal S Kim Y-T Mohanty SK. Ultrafast laser-assisted spatially targeted optoporation into cortical axons and retinal cells in the eye. *J Biomed Opt.* (2017) 22:060504. 10.1117/1.JBO.22.6.060504 PMC5490686 28662241

[180] Wan C Li F Li H. Gene therapy for ocular diseases meditated by ultrasound and microbubbles (Review). *Mol Med Rep.* (2015) 12:4803–14. 10.3892/MMR.2015.4054/HTML 26151686 PMC4581786

[181] Rosazza C Meglic SH Zumbusch A Rols M-P Miklavcic D. Gene electrotransfer: a mechanistic perspective. *Curr Gene Ther.* (2016) 16:98. 10.2174/1566523216666160331130040 27029943 PMC5412002

[182] Johnson CJ Berglin L Chrenek MA Redmond TM Boatright JH Nickerson JM. Technical brief: subretinal injection and electroporation into adult mouse eyes. *Mol Vis.* (2008) 14:2211. 19057658 PMC2593752

[183] Chalberg TW Vankov A Molnar FE Butterwick AF Huie P Calos MP Gene transfer to rabbit retina with electron avalanche transfection. *Invest Ophthalmol Vis Sci.* (2006) 47:4083–90. 10.1167/IOVS.06-0092 16936128

[184] Matsuda T Cepko CL. From the Cover: INAUGURAL ARTICLE by a recently elected academy member:electroporation and RNA interference in the rodent retina in vivo and in vitro. *Proc Natl Acad Sci USA.* (2004) 101:16. 10.1073/PNAS.2235688100 14603031 PMC314130

[185] Touchard E Berdugo M Bigey P El Sanharawi M Savoldelli M Naud MC Suprachoroidal electrotransfer: a nonviral gene delivery method to transfect the choroid and the retina without detaching the retina. *Mol Ther.* (2012) 20:1559. 10.1038/MT.2011.304 22252448 PMC3412485

[186] Dezawa M Takano M Negishi H Mo X Oshitari T Sawada H. Gene transfer into retinal ganglion cells by in vivo electroporation: a new approach. *Micron.* (2002) 33:1–6. 10.1016/S0968-4328(01)00002-6 11473808

[187] ONdrugDelivery. *CREATING SUSTAINABLE BIOTHERAPEUTICS FACTORIES IN THE EYE.* (2018). Available online at: https://ondrugdelivery.com/creating-sustainable-biotherapeutics-factories-eye/ (accessed on March 27, 2022).

[188] Eyevensys. *Technology.* (2022). Available online at: https://www.eyevensys.com/technology/ (accessed on March 27, 2022).

[189] Boshart M Weber F Jahn G Dorsch-Hler K Fleckenstein B Schaffner W. A very strong enhancer is located upstream of an immediate early gene of human cytomegalovirus. *Cell.* (1985) 41:521–30. 10.1016/S0092-8674(85)80025-8 2985280

[190] Hitoshi N Ken-ichi Y Jun-ichi M. Efficient selection for high-expression transfectants with a novel eukaryotic vector. *Gene.* (1991) 108:193–9. 10.1016/0378-1119(91)90434-D 1660837

[191] Schorpp M Jäger R Schellander K Schenkel J Wagner EF Weiher H The human ubiquitin C promoter directs high ubiquitous expression of transgenes in mice. *Nucleic Acids Res.* (1996) 24:1787–8. 10.1093/NAR/24.9.1787 8650001 PMC145851

[192] Lois C Hong EJ Pease S Brown EJ Baltimore D. Germline transmission and tissue-specific expression of transgenes delivered by lentiviral vectors. *Science.* (2002) 295:868–72. 10.1126/SCIENCE.1067081 11786607

[193] Casco-Robles MM Miura T Chiba C. The newt (Cynops pyrrhogaster) RPE65 promoter: molecular cloning, characterization and functional analysis. *Transgenic Res.* (2015) 24:463. 10.1007/S11248-014-9857-1 25490979 PMC4436847

[194] Boulanger A Redmond TM. Expression and promoter activation of the Rpe65 gene in retinal pigment epithelium cell lines. *Curr Eye Res.* (2002) 24:368–75. 10.1076/CEYR.24.5.368.8523 12434305

[195] Ye GJ Budzynski E Sonnentag P Nork TM Sheibani N Gurel Z Cone-specific promoters for gene therapy of achromatopsia and other retinal diseases. *Hum Gene Ther.* (2016) 27:72–82. 10.1089/HUM.2015.130 26603570 PMC4741229

[196] Xiong W Wu DM Xue Y Wang SK Chung MJ Ji X AAV cis-regulatory sequences are correlated with ocular toxicity. *Proc Natl Acad Sci USA.* (2019) 116:5785–94. 10.1073/PNAS.1821000116/-/DCSUPPLEMENTAL 30833387 PMC6431174

[197] Boye SE Alexander JJ Boye SL Witherspoon CD Sandefer KJ Conlon TJ The human rhodopsin kinase promoter in an AAV5 vector confers rod- and cone-specific expression in the primate retina. *Hum Gene Ther.* (2012) 23:1101. 10.1089/HUM.2012.125 22845794 PMC3472519

[198] Pacak CA Sakai Y Thattaliyath BD Mah CS Byrne BJ. Tissue specific promoters improve specificity of AAV9 mediated transgene expression following intra-vascular gene delivery in neonatal mice. *Genet Vaccines Ther.* (2008) 6:13. 10.1186/1479-0556-6-13 18811960 PMC2557000

[199] Hulliger EC Hostettler SM Kleinlogel S. Empowering retinal gene therapy with a specific promoter for human rod and cone on-bipolar cells. *Mol Ther Methods Clin Dev.* (2020) 17:505–19. 10.1016/J.OMTM.2020.03.003/ATTACHMENT/9230B41B-EF75-4F65-B3E9-5A4ABC22393E/MMC1.PDF 32258214 PMC7114634

[200] Nassisi M Mohand-Saïd S Dhaenens CM Boyard F Démontant V Andrieu C Expanding the mutation spectrum in ABCA4: sixty novel disease causing variants and their associated phenotype in a large french Stargardt cohort. *Int J Mol Sci.* (2018) 19:2196. 10.3390/IJMS19082196 30060493 PMC6121640

[201] Cremers FPM Lee W Collin RWJ Allikmets R. Clinical spectrum, genetic complexity and therapeutic approaches for retinal disease caused by ABCA4 mutations. *Prog Retin Eye Res.* (2020) 79:100861. 10.1016/J.PRETEYERES.2020.100861 32278709 PMC7544654

[202] Ahmed ZM Riazuddin S Riazuddin S Wilcox ER. The molecular genetics of Usher syndrome. *Clin Genet.* (2003) 63:431–44. 10.1034/J.1399-0004.2003.00109.X 12786748

[203] Lenassi E Vincent A Li Z Saihan Z Coffey AJ Steele-Stallard HB A detailed clinical and molecular survey of subjects with nonsyndromic USH2A retinopathy reveals an allelic hierarchy of disease-causing variants. *Eur J Hum Genet.* (2015) 23:1318–27. 10.1038/EJHG.2014.283 25649381 PMC4592079

[204] Gao FJ Wang DD Chen F Sun HX Hu FY Xu P Prevalence and genetic–phenotypic characteristics of patients with USH2A mutations in a large cohort of Chinese patients with inherited retinal disease. *Br J Ophthalmol.* (2021) 105:87. 10.1136/BJOPHTHALMOL-2020-315878 32188678 PMC7788223

[205] Marlhens F Bareil C Griffoin JM Zrenner E Amalric P Eliaou C Mutations in RPE65 cause Leber’s congenital amaurosis. *Nat Genet.* (1997) 17:139–41. 10.1038/NG1097-139 9326927

[206] Schimmer J Breazzano S. Investor outlook: significance of the positive LCA2 gene therapy phase III results. *Hum Gene Ther Clin Dev.* (2015) 26:208–10. 10.1089/HUMC.2015.29004.SCH 26684444

[207] Bennett J. Taking stock of retinal gene therapy: looking back and moving forward. *Mol Ther.* (2017) 25:1076–94. 10.1016/J.YMTHE.2017.03.008 28391961 PMC5417792

[208] Bainbridge JW Ali RR. Success in sight: the eyes have it! Ocular gene therapy trials for LCA look promising. *Gene Ther.* (2008) 15:1191–2. 10.1038/GT.2008.117 18711389

[209] Dryja TP McGee TL Reichel E Hahn LB Cowley GS Yandell DW A point mutation of the rhodopsin gene in one form of retinitis pigmentosa. *Nature.* (1990) 343:364–6. 10.1038/343364A0 2137202

[210] Daiger SP Bowne SJ Sullivan LS. Genes and mutations causing autosomal dominant retinitis pigmentosa. *Cold Spring Harb Perspect Med.* (2014) 5:a017129. 10.1101/CSHPERSPECT.A017129 25304133 PMC4588133

[211] Ham M Han J Osann K Smith M Kimonis V. Meta-analysis of genotype-phenotype analysis of OPA1 mutations in autosomal dominant optic atrophy. *Mitochondrion.* (2019) 46:262–9. 10.1016/J.MITO.2018.07.006 30165240

[212] Jurkute N Majander A Bowman R Votruba M Abbs S Acheson J Clinical utility gene card for: inherited optic neuropathies including next-generation sequencing-based approaches. *Eur J Hum Genet.* (2019) 27:494. 10.1038/S41431-018-0235-Y 30143805 PMC6460557

[213] Cunha DL Arno G Corton M Moosajee M. The spectrum of PAX6 mutations and genotype-phenotype correlations in the eye. *Genes (Basel).* (2019) 10:1050. 10.3390/GENES10121050 31861090 PMC6947179

[214] Farrar GJ Millington-Ward S Chadderton N Humphries P Kenna PF. Gene-based therapies for dominantly inherited retinopathies. *Gene Ther.* (2012) 19:137–44. 10.1038/GT.2011.172 22089493

[215] Giannelli SG Luoni M Castoldi V Massimino L Cabassi T Angeloni D Cas9/sgRNA selective targeting of the P23H Rhodopsin mutant allele for treating retinitis pigmentosa by intravitreal AAV9.PHP.B-based delivery. *Hum Mol Genet.* (2018) 27:761–79. 10.1093/HMG/DDX438 29281027

[216] Audo I Manes G Mohand-Saïd S Friedrich A Lancelot ME Antonio A Spectrum of rhodopsin mutations in french autosomal dominant rod–cone dystrophy patients. *Invest Ophthalmol Vis Sci.* (2010) 51:3687–700. 10.1167/IOVS.09-4766 20164459 PMC3102265

[217] Lewin AS Rossmiller B Mao H. Gene augmentation for adRP mutations in RHO. *Cold Spring Harb Perspect Med.* (2014) 4:a017400. 10.1101/CSHPERSPECT.A017400 25037104 PMC4143106

[218] Li P Kleinstiver BP Leon MY Prew MS Navarro-Gomez D Greenwald SH Allele-specific CRISPR-Cas9 genome editing of the single-base P23H mutation for rhodopsin-associated dominant retinitis pigmentosa. *Cris J.* (2018) 1:55–64. 10.1089/CRISPR.2017.0009 31021187 PMC6319323

[219] Kurata K Hosono K Hayashi T Mizobuchi K Katagiri S Miyamichi D X-linked retinitis pigmentosa in japan: clinical and genetic findings in male patients and female carriers. *Int J Mol Sci.* (2019) 20:1518. 10.3390/IJMS20061518 30917587 PMC6470860

[220] Mitsios A Dubis AM Moosajee M. Choroideremia: from genetic and clinical phenotyping to gene therapy and future treatments. *Ther Adv Ophthalmol.* (2018) 10:251584141881749. 10.1177/2515841418817490 30627697 PMC6311551

[221] Daiger SP Bowne SJ Sullivan LS. Perspective on genes and mutations causing retinitis pigmentosa. *Arch Ophthalmol.* (2007) 125:151–8. 10.1001/ARCHOPHT.125.2.151 17296890 PMC2580741

[222] Ebenezer ND Michaelides M Jenkins SA Audo I Webster AR Cheetham ME Identification of novel RPGR ORF15 mutations in x-linked progressive cone-rod dystrophy (XLCORD) families. *Invest Ophthalmol Vis Sci.* (2005) 46:1891–8. 10.1167/IOVS.04-1482 15914600

[223] Ng D Thakker N Corcoran CM Donnai D Perveen R Schneider A Oculofaciocardiodental and Lenz microphthalmia syndromes result from distinct classes of mutations in BCOR. *Nat Genet.* (2004) 36:411–6. 10.1038/NG1321 15004558

[224] Moosajee M Ali MA Wong SC. Retinal angiography findings in male infant with incontinentia pigmenti and sickle cell trait. *JAMA Ophthalmol.* (2018) 136:e183140. 10.1001/JAMAOPHTHALMOL.2018.3140 30418511

[225] Maguire AM Simonelli F Pierce EA Pugh EN Mingozzi F Bennicelli J Safety and efficacy of gene transfer for Leber’s congenital amaurosis. *N Engl J Med.* (2008) 358:2240–8. 10.1056/NEJMOA0802315 18441370 PMC2829748

[226] Maguire AM High KA Auricchio A Wright JF Pierce EA Testa F Age-dependent effects of RPE65 gene therapy for Leber’s congenital amaurosis: a phase 1 dose-escalation trial. *Lancet (London, England).* (2009) 374:1597–605. 10.1016/S0140-6736(09)61836-5 PMC4492302 19854499

[227] Zeitz C Robson AG Audo I. Congenital stationary night blindness: an analysis and update of genotype-phenotype correlations and pathogenic mechanisms. *Prog Retin Eye Res.* (2015) 45:58–110. 10.1016/J.PRETEYERES.2014.09.001 25307992

[228] Cremers FPM Boon CJF Bujakowska K Zeitz C. Special issue introduction: inherited retinal disease: novel candidate genes, genotype-phenotype correlations, and inheritance models. *Genes (Basel).* (2018) 9:215. 10.3390/GENES9040215 29659558 PMC5924557

[229] Sahel JA Newman NJ Yu-Wai-Man P Vignal-Clermont C Carelli V Biousse V Gene therapies for the treatment of leber hereditary optic neuropathy. *Int Ophthalmol Clin.* (2021) 61:195–208. 10.1097/IIO.0000000000000364 34584057 PMC8478322

[230] Sahel JA Roska B. Gene therapy for blindness. *Annu Rev Neurosci.* (2013) 36:467–88. 10.1146/ANNUREV-NEURO-062012-170304 23724995

[231] Sahel JA Léveillard T Picaud S Dalkara D Marazova K Safran A Functional rescue of cone photoreceptors in retinitis pigmentosa. *Graefes Arch Clin Exp Ophthalmol.* (2013) 251:1669–77. 10.1007/S00417-013-2314-7 23575948

[232] Simon CJ Sahel JA Duebel J Herlitze S Dalkara D. Opsins for vision restoration. *Biochem Biophys Res Commun.* (2020) 527:325–30. 10.1016/J.BBRC.2019.12.117 31982136

[233] Quinn N Csincsik L Flynn E Curcio CA Kiss S Sadda SVR The clinical relevance of visualising the peripheral retina. *Prog Retin Eye Res.* (2019) 68:83–109. 10.1016/J.PRETEYERES.2018.10.001 30316018

[234] Pichi F Morara M Veronese C Nucci P Ciardella AP. Multimodal imaging in hereditary retinal diseases. *J Ophthalmol.* (2013) 2013:11. 10.1155/2013/634351 23710333 PMC3655643

[235] De Silva SR Arno G Robson AG Fakin A Pontikos N Mohamed MD The X-linked retinopathies: Physiological insights, pathogenic mechanisms, phenotypic features and novel therapies. *Prog Retin Eye Res.* (2021) 82:100898. 10.1016/J.PRETEYERES.2020.100898 32860923

[236] MacLaren RE Groppe M Barnard AR Cottriall CL Tolmachova T Seymour L Retinal gene therapy in patients with choroideremia: initial findings from a phase 1/2 clinical trial. *Lancet.* (2014) 383:1129. 10.1016/S0140-6736(13)62117-0 PMC4171740 24439297

[237] Lazow MA Hood DC Ramachandran R Burke TR Wang YZ Greenstein VC Transition zones between healthy and diseased retina in choroideremia (CHM) and stargardt disease (STGD) as compared to retinitis pigmentosa (RP). *Invest Ophthalmol Vis Sci.* (2011) 52:9581. 10.1167/IOVS.11-8554 22076985 PMC3341121

[238] Huang XF Huang F Wu KC Wu J Chen J Pang CP Genotype-phenotype correlation and mutation spectrum in a large cohort of patients with inherited retinal dystrophy revealed by next-generation sequencing. *Genet Med.* (2015) 17:271–8. 10.1038/GIM.2014.138 25356976

[239] McKibbin M Ali M Mohamed MD Booth AP Bishop F Pal B Genotype-phenotype correlation for leber congenital amaurosis in Northern Pakistan. *Arch Ophthalmol.* (2010) 128:107–13. 10.1001/ARCHOPHTHALMOL.2010.309 20065226

[240] Jiang J Wu X Shen D Dong L Jiao X Hejtmancik JF Analysis of RP2 and RPGR mutations in five X-linked chinese families with retinitis pigmentosa. *Sci Rep.* (2017) 7:44465. 10.1038/srep44465 28294154 PMC5353642

[241] Nebbioso M Franzone F Lambiase A La Cava M Mallone F Pizzuti A X-linked dominant RPGR gene mutation in a familial Coats angiomatosis. *BMC Ophthalmol.* (2021) 21:37. 10.1186/S12886-020-01791-5 33446141 PMC7807486

[242] Churchill JD Bowne SJ Sullivan LS Lewis RA Wheaton DK Birch DG Mutations in the X-linked retinitis pigmentosa genes RPGR and RP2 found in 8.5% of families with a provisional diagnosis of autosomal dominant retinitis pigmentosa. *Invest Ophthalmol Vis Sci.* (2013) 54:1411–6. 10.1167/IOVS.12-11541 23372056 PMC3597192

[243] Al-Maskari A O’Grady A Pal B McKibbin M. Phenotypic progression in X-linked retinitis pigmentosa secondary to a novel mutation in the RPGR gene. *Eye (Lond).* (2009) 23:519–21. 10.1038/EYE.2008.427 19218993

[244] Vaclavik V Gaillard MC Tiab L Schorderet DF Munier FL. Variable phenotypic expressivity in a Swiss family with autosomal dominant retinitis pigmentosa due to a T494M mutation in the PRPF3 gene. *Mol Vis.* (2010) 16:467. 20309403 PMC2842095

[245] Mandai M Fujii M Hashiguchi T Sunagawa GA Ito S Sun J iPSC-derived retina transplants improve vision in rd1 end-stage retinal-degeneration mice. *Stem Cell Rep.* (2017) 8:69–83. 10.1016/J.STEMCR.2016.12.008 28076757 PMC5233464

[246] Morizur L Herardot E Monville C Ben M’Barek K. Human pluripotent stem cells: a toolbox to understand and treat retinal degeneration. *Mol Cell Neurosci.* (2020) 107:103523. 10.1016/J.MCN.2020.103523 32634576

[247] Wang Y Tang Z Gu P. Stem/progenitor cell-based transplantation for retinal degeneration: a review of clinical trials. *Cell Death Dis.* (2020) 11:793. 10.1038/s41419-020-02955-3 32968042 PMC7511341

[248] Martinelli I Tayebati SK Tomassoni D Nittari G Roy P Amenta F. Brain and retinal organoids for disease modeling: the importance of in vitro blood-brain and retinal barriers studies. *Cells.* (2022) 11:1120. 10.3390/CELLS11071120 35406683 PMC8997725

[249] Schwartz SD Hubschman JP Heilwell G Franco-Cardenas V Pan CK Ostrick RM Embryonic stem cell trials for macular degeneration: a preliminary report. *Lancet (London, England).* (2012) 379:713–20. 10.1016/S0140-6736(12)60028-2 22281388

[250] Schwartz SD Regillo CD Lam BL Eliott D Rosenfeld PJ Gregori NZ Human embryonic stem cell-derived retinal pigment epithelium in patients with age-related macular degeneration and Stargardt’s macular dystrophy: follow-up of two open-label phase 1/2 studies. *Lancet (London, England).* (2015) 385:509–16. 10.1016/S0140-6736(14)61376-3 25458728

[251] Schwartz SD Tan G Hosseini H Nagiel A. Subretinal transplantation of embryonic stem cell-derived retinal pigment epithelium for the treatment of macular degeneration: an assessment at 4 years. *Invest Ophthalmol Vis Sci.* (2016) 57:ORSFc1–9. 10.1167/IOVS.15-18681 27116660

[252] Song WK Park KM Kim HJ Lee JH Choi J Chong SY Treatment of macular degeneration using embryonic stem cell-derived retinal pigment epithelium: preliminary results in Asian patients. *Stem Cell Rep.* (2015) 4:860–72. 10.1016/J.STEMCR.2015.04.005 25937371 PMC4437471

[253] Garber K. RIKEN suspends first clinical trial involving induced pluripotent stem cells. *Nat Biotechnol.* (2015) 33:890–1. 10.1038/NBT0915-890 26348942

[254] Miller AD. Human gene therapy comes of age. *Nature* (1992) 357:455–60. 10.1038/357455A0 1608446

[255] Mitchell P Liew G Gopinath B Wong TY. Age-related macular degeneration. *Lancet (London, England).* (2018) 392:1147–59. 10.1016/S0140-6736(18)31550-2 30303083

[256] Haffner ME. The food and drug administration’s office of orphan products development: incentives, grants, and special designations speed therapies for orphan diseases. *Retina.* (2005) 25 (Suppl. 8):S89–90. 10.1097/00006982-200512001-00045 16374359

